# MicroED characterization of a robust cationic σ-alkane complex stabilized by the [B(3,5-(SF_5_)_2_C_6_H_3_)_4_]^−^ anion, *via* on-grid solid/gas single-crystal to single-crystal reactivity†

**DOI:** 10.1039/d2dt00335j

**Published:** 2022-02-03

**Authors:** Laurence R. Doyle, Emily A. Thompson, Arron L. Burnage, Adrian C. Whitwood, Huw T. Jenkins, Stuart A. Macgregor, Andrew S. Weller

**Affiliations:** Department of Chemistry, University of York Heslington York YO10 5DD UK huw.jenkins@york.ac.uk andrew.weller@york.ac.uk; Institute of Chemical Sciences, Heriot-Watt University Edinburgh EH14 4AS UK S.A.Macgregor@hw.ac.uk

## Abstract

Microcrystalline (∼1 μm) [Rh(Cy_2_PCH_2_CH_2_PCy_2_)(norbornadiene)][S-BAr^F^_4_], [S-BAr^F^_4_] = [B(3,5-(SF_5_)_2_C_6_H_3_)_4_]^−^, reacts with H_2_ in a single-crystal to single-crystal transformation to form the σ-alkane complex [Rh(Cy_2_PCH_2_CH_2_PCy_2_)(norbornane)][S-BAr^F^_4_], for which the structure was determined by microcrystal Electron Diffraction (microED), to 0.95 Å resolution, *via* an on-grid hydrogenation, and a complementary single-crystal X-ray diffraction study on larger, but challenging to isolate, crystals. Comparison with the [BAr^F^_4_]^−^ analogue [Ar^F^ = 3,5-(CF_3_)_2_(C_6_H_3_)] shows that the [S-BAr^F^_4_]^−^ anion makes the σ-alkane complex robust towards decomposition both thermally and when suspended in pentane. Subsequent reactivity with dissolved ethene in a pentane slurry, forms [Rh(Cy_2_PCH_2_CH_2_PCy_2_)(ethene)_2_][S-BAr^F^_4_], and the catalytic dimerisation/isomerisation of ethene to 2-butenes. The increased stability of [S-BAr^F^_4_]^−^ salts is identified as being due to increased non-covalent interactions in the lattice, resulting in a solid-state molecular organometallic material with desirable stability characteristics.

## Abstract 2

The thermally and chemically robust σ-alkane complex [Rh(Cy_2_PCH_2_CH_2_PCy_2_)(norborane)][B(3,5-(SF_5_)_2_C_6_H_3_)_4_] is characterized by micro-electron diffraction using on-grid single-crystal to single-crystal reactivity.

## Graphical abstract

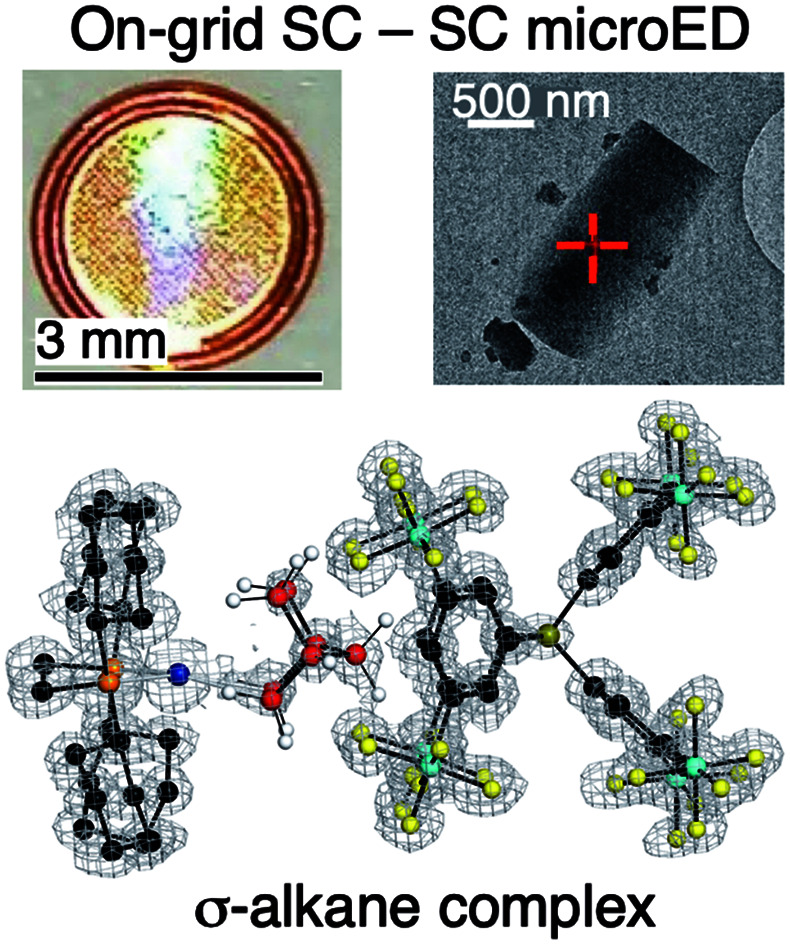

## Introduction

**Scheme 1:**
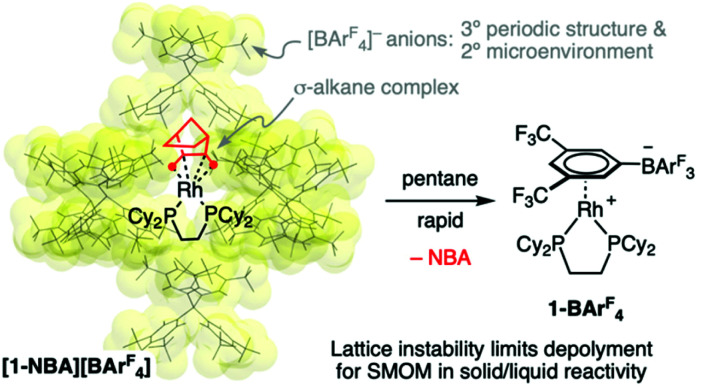
Arrangement of [BAr^F^_4_]^−^ anions in [1-NBA][BAr^F^_4_] and its decomposition when single crystals are suspended in pentane.

Single-crystal to single-crystal (SC–SC) transformations allow for the structural characterisation of highly reactive organometallic species *in crystallo*,^1–3^ by the application of an external agent (*e.g.*, reactants^4,5^ or UV-light^6^) that leads to a reaction at the metal site. For solid-state molecular organometallic (SMOM) systems, that are non-framework crystalline materials,^2^ we have been developing SC–SC techniques to synthesise, characterize, and exploit in onward reactivity, σ-alkane complexes – species that have only a transient existence in solution at low temperatures.^7–9^ This approach is exemplified by the solid/gas SC–SC reaction of a precursor diene complex [Rh(Cy_2_PCH_2_CH_2_PCy_2_)(NBD)][BAr^F^_4_], [1-NBD][BAr^F^_4_] [NBD = norbornadiene; Ar^F^ = 3,5-(CF_3_)_2_(C_6_H_3_)], with H_2_ that results in the isolation of the corresponding σ-alkane complex [Rh(Cy_2_PCH_2_CH_2_PCy_2_)(NBA)][BAr^F^_4_], [1-NBA][BAr^F^_4_] (NBA = norbornane).^10^ The [BAr^F^_4_]^−^ anions form an octahedral motif around each cation that provides a 3°-periodic molecular framework and 2°-stabilising non-covalent interactions that collectively support the weak 3c–2e binding of the alkane with the metal centre, Scheme 1.^11^ This σ-alkane complex also undergoes further solid–gas SC–SC reactivity with alkenes by simple displacement of NBA.^12,13^ However suspending crystalline [1-NBA][BAr^F^_4_] in pentane results in rapid loss of NBA, and coordination of the [BAr^F^_4_]^−^ anion to form the pentane-soluble zwitterion 1-BAr^F^_4_.^10^ So while the microenvironment provided by the [BAr^F^_4_]^−^ anions supports the isolation and characterisation of σ-alkane complexes they are not robust towards solid/liquid reactivity, a limitation in synthesis and catalysis. This is in contrast with extended framework materials such as metal organic frameworks, MOFs, for which suitable metal/linker combinations can promote chemical and thermal resilience.^14^ MOFs are also well documented to undergo SC–SC transformations using both solid/gas and solid/liquid reactivity.^15,16^

**Scheme 2:**
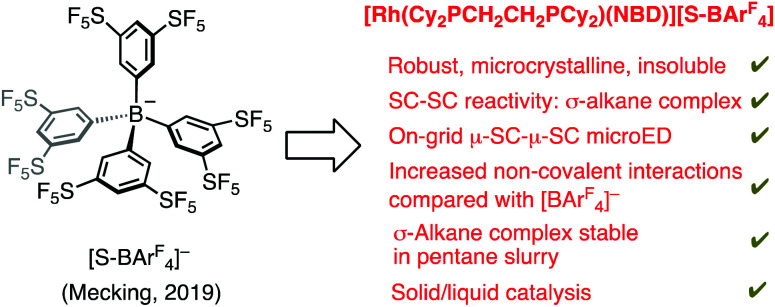
This work.

We now report (Scheme 2) that by changing the anion in [1-NBA][BAr^F^_4_] to Mecking's, recently reported, [B(3,5-(SF_5_)_2_C_6_H_3_)_4_]^−^ anion ([S-BAr^F^_4_])^17^ increased C–H^*δ*+^⋯ ^*δ*−^F–C non-covalent interactions^11^ in the lattice lead to a robust, microcrystalline, σ-alkane complex, that shows improved stability characteristics under both thermal and solid/liquid reactivity conditions. This allows for catalytic ethene dimerisation/isomerisation in a pentane slurry without decomposition to the zwitterion. The structural characterisation of this σ-alkane complex was aided by on-grid solid/gas SC–SC microcrystal Electron Diffraction (microED) methods,^18,19^ complemented by a single-crystal to single-crystal X-ray diffraction study on larger, but difficult to obtain, crystals.

## Results and discussion

**Fig. 1:**
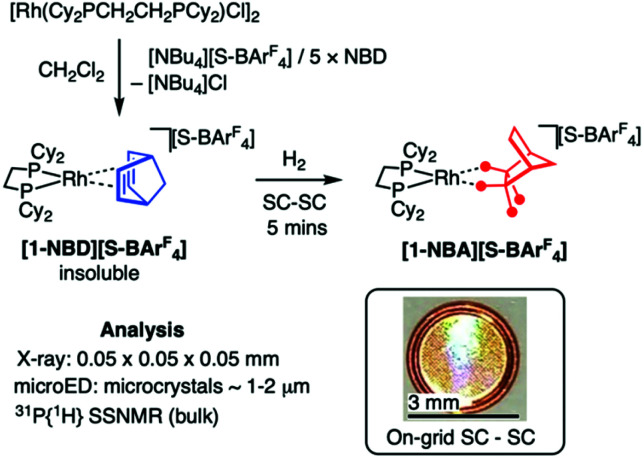
Synthesis of [1-NBA][S-BAr^F^_4_] by SC–SC techniques. Inset shows TEM grid used for on-grid μ-SC to μ-SC.

Addition of sparingly soluble [NBu_4_][S-BAr^F^_4_]^17^ to [Rh(Cy_2_PCH_2_CH_2_PCy_2_)Cl]_2_ in CH_2_Cl_2_ solvent, with excess NBD, leads to the precipitation of analytically pure microcrystals (∼1 to 10 μm) of [Rh(Cy_2_PCH_2_CH_2_PCy_2_)(NBD)][S-BAr^F^_4_], [1-NBD][S-BAr^F^_4_], Fig. 1. Changing the solvent to acetone or THF resulted in the same microcrystalline solid. ^31^P{^1^H} and ^13^C{^1^H} SSNMR (solid-state NMR) data were consistent with the formulation of [1-NBD][S-BAr^F^_4_], with a broad signal observed at *δ* 75.7 in the former [*cf*. [1-NBD][BAr^F^_4_]*δ* 76 (ref. 10], and two diagnostic signals in the alkene region in the latter [*δ* 91.2, 84.1].^20^ While the insolubility of [1-NBD][S-BAr^F^_4_] suggested desirable robustness, it counterpoints that of [Ni(allyl)(mesitylene)][S-BAr^F^_4_]^17^ and [1-NBD][BAr^F^_4_]^10^ which are soluble in CH_2_Cl_2_.

**Fig. 2:**
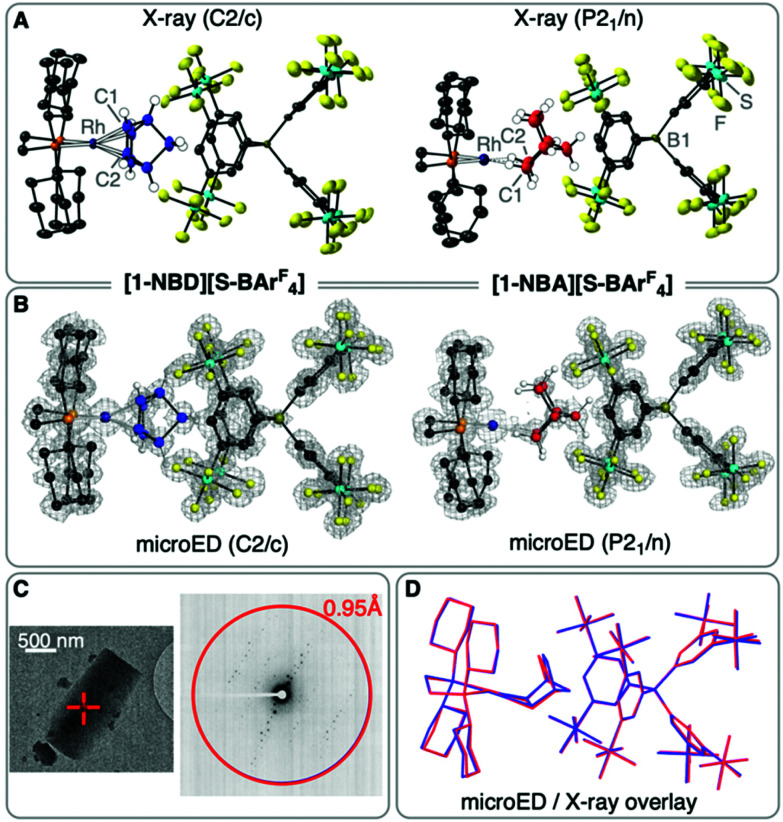
(A) SC X-ray (50% displacement ellipsoids and selected H-atoms) of [1-NBD][S-BAr^F^_4_] and [1-NBA][S-BAr^F^_4_]. (B) microED structures (ball and stick including selected H-atoms, electrostatic potential map) of [1-NBD][S-BAr^F^_4_] and [1-NBA][S-BAr^F^_4_]. (C) Example of selected crystals for microED and diffraction pattern. (D) Overlay of SC X-ray (blue) and microED (red) structures of [1-NBA][S-BAr^F^_4_].

Larger (0.05 mm^3^) crystals of [1-NBD][S-BAr^F^_4_]^21^ suitable for a SC X-ray diffraction study could be formed from a slower crystallisation from hot CH_2_Cl_2_ (sealed vessel, 40 °C). However, this method was unpredictable and, frustratingly, nearly always resulted in microcrystalline material. A resulting structural solution is isomorphous with [1-NBD][BAr^F^_4_]^10^ showing a pseudo square planar [1-NBD]^+^ cation, sitting on a crystallographic *C*_2_-axis, with the NBD ligand located in the cleft formed by two anion aryl rings (Fig. 2A), and an ∼*O*_h_ arrangement of [S-BAr^F^_4_]^−^ anions around the cation (Fig. S19†).

As with the [BAr^F^_4_]^−^ analogue, addition of H_2_ (1.3 atm) to single crystals of [1-NBD][S-BAr^F^_4_],^10^ resulted in a fast (5 min) SC–SC solid/gas reaction to give the σ-alkane complex [1-NBA][S-BAr^F^_4_], Fig. 2A [*R*(2*σ*) = 8.7%]. The gross structural change on hydrogenation of NBD to form the alkane NBA is a rotation by ∼90°, and a lengthening of the key C–C bonds [*e.g.*, C1–C2, 1.60(1) Å]. There is an associated change in space group^10,22^ from *C*2/*c* to *P*2_1_/*n*. The alkane ligand binds with the Rh(i) centre through two *endo* C–H⋯Rh 3c–2e bonds, as signalled by the Rh⋯C distances: 2.41(1), 2.42(1) Å.^9^ Hydrogen atoms were placed in calculated positions, and periodic DFT calculations (see later) confirm a 1,2-η^2^,η^2^ binding mode, as for [1-NBA][BAr^F^_4_].^10^ The ^31^P{^1^H} SSNMR spectrum of powdered [1-NBA][S-BAr^F^_4_] shows this reaction to be quantitative, by a downfield-shifted apparent triplet being observed at *δ* 110.1 [*J*(RhP) = 196 Hz], consistent with the formation of a σ-alkane complex.^10^[1-NBA][S-BAr^F^_4_] is unchanged after 3 weeks under Ar. These data combine to show that the [S-BAr^F^_4_]^−^ anion supports the formation of a stable σ-alkane complex, unlike alternative anions such as [B(3,5-Cl_2_-C_6_H_3_)_4_]^−^ where loss of NBA and anion-coordination results.^23^

As the formation of suitably sized crystals for SC X-ray diffraction was challenging and stochastic, we were interested if microED could provide a complementary structural solution of [1-NBA][S-BAr^F^_4_]. MicroED has recently been used for the structural determination of microcrystalline organometallic^24^ and coordination complexes,^25^ as well as complex organic molecules^19,26^ and MOFs.^27^ Suitably thin crystals (≲300 nm) of size ∼1–2 μm could be reliably produced by crushing of precursor microcrystalline [1-NBD][S-BAr^F^_4_] and transfer to a TEM grid. Continuous rotation data were collected at low temperature (80 K), using a very low flux 200 keV electron beam (∼0.01 e^−^ Å^−2^ s^−1^). Reduction and merging of data collected from 4 crystals provided a high completeness dataset (96.6%, 0.95 Å resolution). Refinement, using anisotropic displacement parameters, resulted in a good structural solution, Fig. 2B (*R*_1_ = 15.7%, *C*2/*c* space group). Mapping this structure onto that from the SC X-ray showed excellent fidelity between the two (Fig. S19†).

With this microED structure in hand for [1-NBD][S-BAr^F^_4_] we developed an on-grid methodology for microcrystalline SC to SC solid/gas hydrogenation to form [1-NBA][S-BAr^F^_4_]. This involved depositing finely powdered microcrystalline [1-NBD][S-BAr^F^_4_] on a Cu TEM grid with holey carbon support film (Fig. 1 inset), addition of H_2_ (1.3 atm for 5 minutes), and transportation to the cryo-TEM under an Ar atmosphere. Rapid transfer of the grid to the loading cassette under a N_2_ vapour blanket and then cooling to 80 K allowed for compositional integrity of the sample to be maintained.^28^ Analysis provided a good quality combined dataset (94.0% completeness, 0.95 Å resolution) from 9 crystals. The resulting structural refinement (*R*_1_ = 16.4%, *P*2_1_/*n* space group), Fig. 2B, maps well with the SC X-ray structure, Fig. 2D, unequivocally demonstrating the formation of the σ-alkane complex [1-NBA][S-BAr^F^_4_]. The use of microED for μ-SC to μ-SC transformations for on-grid solid/gas reactivity, to our knowledge, has not been reported. *In situ*, on-grid topochemical polymerizations have been recently described.^29^

**Fig. 3:**
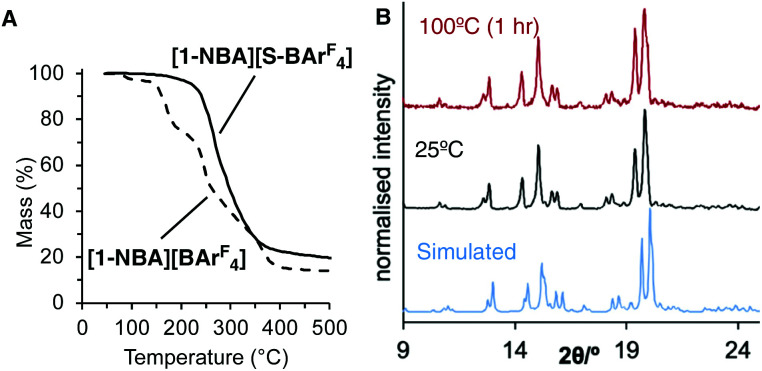
(A) TGA traces (ramp 10 °C min^−1^) of [1-NBA][S-BAr^F^_4_] and [1-NBA][BAr^F^_4_]. (B) Powder X-ray diffraction data for [1-NBA][S-BAr^F^_4_].

Microcrystalline [1-NBA][S-BAr^F^_4_] can be suspended in pentane, returning unreacted sample after 2 hours (^31^P{^1^H} SSNMR). This contrasts with [1-NBA][BAr^F^_4_] where the zwitterion 1-BAr^F^_4_ is formed by displacement of NBA, Scheme 1.^30^ This difference in stability is carried over to the thermal mass loss behavior of the two σ-alkane complexes. TGA shows that [1-NBA][S-BAr^F^_4_] only starts to undergo significant mass loss at higher temperatures (170 °C) compared with [1-NBA][BAr^F^_4_] (80 °C), Fig. 3A. Reflecting this, powder X-ray diffraction data of a sample of [1-NBA][S-BAr^F^_4_] heated for 1 hour at 100 °C under Ar remains unchanged, Fig. 3B, whereas [1-NBA][BAr^F^_4_] immediately decomposes to form an oil.

**Scheme 3:**
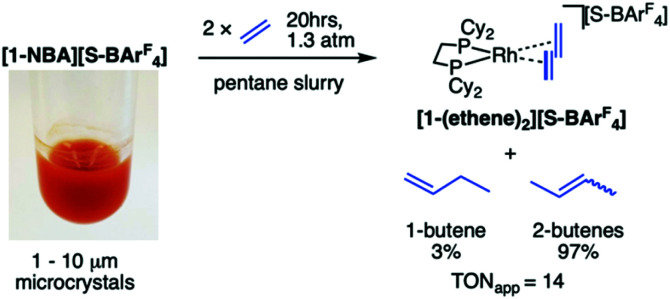
Dimerization of ethene and the formation of [1-(ethene)_2_][S-BAr^F^_4_] in a pentane suspension.

Leveraging the insolubility in pentane, a heterogeneous solid/liquid reaction of [1-NBA][S-BAr^F^_4_] with excess ethene (1.3 atm, [Rh]_TOTAL_ = 7 mol%) results in the displacement of NBA by ethene and the formation of [Rh(Cy_2_PCH_2_CH_2_PCy_2_)(η^2^-H_2_C=CH_2_)_2_][S-BAr^F^_4_], [1-(ethene)_2_][S-BAr^F^_4_], (Scheme 3). This complex was characterised by ^31^P{^1^H} and ^31^C{^1^H} SSNMR spectroscopy, and by a SC–SC solid/gas study on larger crystals (0.05 mm^3^, Fig. S22 †^31^), and the resulting data are very similar to [1-(ethene)_2_][BAr^F^_4_].^12^ A thermodynamic mixture^32^ of 1-butene and *cis*/*trans*-2-butenes (3 : 97 respectively) is also formed. Recharging with ethene restarted catalysis, giving an overall modest TON_app_ of ∼14 (ToF_app_ ∼ 0.4 h^−1^). [1-NBA][S-BAr^F^_4_] was independently shown to isomerise 1-butene to 2-butenes in a pentane slurry, suggesting a cascade process of ethene dimerisation to 1-butene,^33^ followed by isomerisation (as has been shown in solid/gas reactivity of [1-NBA][BAr^F^_4_] with 1-butene^12^). The filtered supernatant above [1-(ethene)_2_][S-BAr^F^_4_] was inactive, demonstrating a heterogeneous catalyst with no leaching of soluble catalyst. We suggest the conditions of a pentane slurry facilitate the ingress of reactants (ethene) and egress of products (butenes) from the surface of the crystal, that retains [1-(ethene)_2_][S-BAr^F^_4_] as the final resting state, rather than a butene complex.^12,34^ While the catalytic activity is modest in comparison with catalyst-functionalized MOFs, that can promote very high ToF for ethene dimerization, *e.g.* ∼10^4^ h^−1^ under slurry conditions,^35^ the current study demonstrates the potential for solid/liquid reactivity in non-framework crystalline SMOM materials. The underlying reasons for this stability were explored using computational methods.

**Fig. 4:**
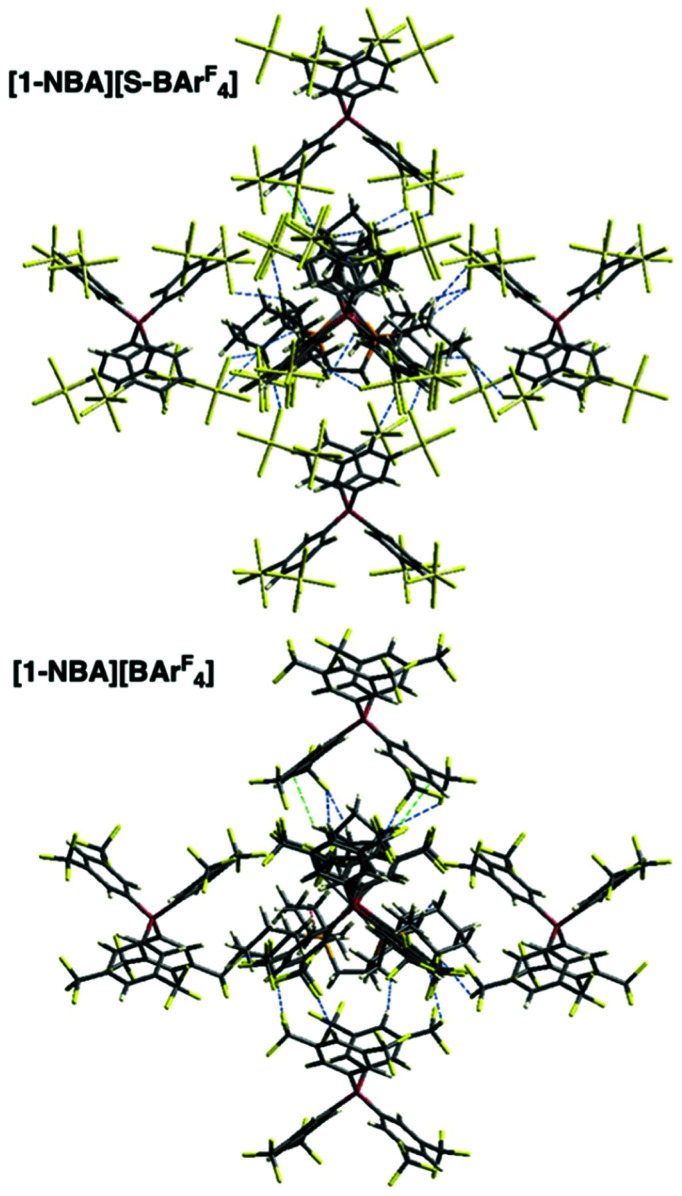
(A) Short contacts between the [1-NBA]^+^ cations and the surrounding anions in [1-NBA][S-BAr^F^_4_] and [1-NBA][BAr^F^_4_].

Full optimisation of the structure of [1-NBA][S-BAr^F^_4_] in the solid state using periodic DFT calculations provided excellent agreement with the SC X-ray structure and electronic structure analyses (QTAIM, NBO and NCI plots) confirmed a 1,2-η^2^,η^2^ binding mode for the NBA ligand, very similar to [1-NBA][BAr^F^_4_] (Fig. S23–25†). These similarities extend to the alkane binding energies (Table 1) with very close values obtained for both the solid-state incorporation energies (Δ*E*_1_) and the molecular interaction energy^11^ (Δ*E*_2_) (Table 1). The additional contribution to alkane binding from the solid state microenvironment (Δ*E*_3_) is therefore only marginally higher for [1-NBA][S-BAr^F^_4_]. In contrast the computed normalised lattice energy is 19.7 kcal mol^−1^ (or 16%) higher in [1-NBA][S-BAr^F^_4_]. As this is not due to any intrinsic increase in the alkane binding energy, this must reflect a greater stabilisation of the 3°-periodic molecular framework in this species. Indeed, the [1-NBA]^+^ cation in [1-NBA][S-BAr^F^_4_] shows many more C–F⋯H–C close contacts at or below the sum of the van der Waals radii compared to [1-NBA][BAr^F^_4_], Fig. 4, S28.† Closer analysis reveals that contacts involving the NBA ligand are similar for both structures (consistent with the similar alkane binding energies). However, many additional contacts between the cation and the anions around the equatorial belt of the *O*_h_ anion environment are seen for [1-NBA][S-BAr^F^_4_].

**Table 1:** Computed binding energies (kcal mol^−1^) for [1-NBA][X] species (X = S-BAr^F^_4_ and BAr^F^_4_). Data for X = BAr^F^_4_ are from Reference^11^

Quantity	X = S-BArF_4_	X = BArF_4_
Normalised lattice energy^a^	139.4	119.7
Incorporation energy, Δ*E*_1 _b	−48.5	−47.1
Molecular interaction energy, Δ*E*_2 _c	−33.0	−33.1
Microenvironment stabilisation energy, Δ*E*_3 _d	−15.5	−14.0

a Computed lattice energy/*Z.*

b Energy to remove one NBA from the [1-NBA][X] unit cell.

c Energy to remove NBA from an isolated [1-NBA]^+^ cation.

d Δ*E*_1_ − Δ*E*_2._

## Conclusions

Increasing the number of stabilising non-covalent interactions in the anion framework leads to more robust crystalline SMOM materials, both allowing for stability under solid/liquid catalysis conditions and SC–SC reactivity to form a σ-alkane complex. The parallel development of on-grid SC to SC methods for microED extends the use of this technique^18,19,24^ towards intrinsically reactive microcrystalline organometallics. It will be interesting to see if these combined developments can be harnessed to synthesise organometallic complexes with even more weakly binding ligands than the alkane norbornane.

## Author contributions

LRD, EAT, ACW and HTJ designed and performed experiments; ALB performed all DFT calculations and analyses; HTJ, SAM and ASW wrote the manuscript with input from all the authors.

## Conflicts of interest

There are no conflicts to declare.

## Supplementary Material

DT-051-D2DT00335J-s001

DT-051-D2DT00335J-s002
