# Supplementary material for: MicroED characterization of a robust cationic σ-alkane complex stabilized by the [B(3,5-(SF5)2C6H3)4]− anion, via on-grid solid/gas single-crystal to single-crystal reactivity
Source: Dalton Trans. 2022 Feb 3;51(9):3661–5. doi: 10.1039/d2dt00335j (PMC8902584; doi:10.1039/d2dt00335j)
Supplement: DT-051-D2DT00335J-s001 [file DT-051-D2DT00335J-s001.pdf]

Electronic Supplementary Material (ESI) for Dalton Transactions.
This journal is © The Royal Society of Chemistry 2022

Supporting Information

MicroED Characterization of a Robust Cationic σ-Alkane Complex Stabilized by the $[B(3,5-(SF_5)_2C_6H_3)_4]^-$ Anion, via On-Grid Solid/Gas Single-Crystal to Single-Crystal Reactivity.

Laurence R. Doyle,[a] Emily A. Thompson,[a] Arron L. Burnage,[b] Adrian C. Whitwood,[a] Huw T. Jenkins,*[a] Stuart A. Macgregor,*[b] Andrew S. Weller.*[a]

[a] Department of Chemistry, University of York, Heslington, York YO10 5DD (UK)

[b] Institute of Chemical Sciences, Heriot Watt University, Edinburgh EH14 4AS (UK)

**S.1**	**EXPERIMENTAL DETAILS**		S3
**S.2**	**SYNTHETIC PROCEDURES**		S4
	**S.2.1**	Synthesis of **[1-NBD][S-BAr$^F_4$]**	S4
	**S.2.2**	Synthesis of **[1-NBA][S-BAr$^F_4$]**	S5
	**S.2.3**	Synthesis of **endo-d$_4$-[1-NBA][S-BAr$^F_4$]**, **exo-d$_4$-[1-NBA][S-BAr$^F_4$]**, and **d$_8$-[1-NBA][S-BAr$^F_4$]**	S8
	**S.2.4**	Stability of **[1-NBA][S-BAr$^F_4$]** and **[1-NBA][BAr$^F_4$]** in pentane	S10
	**S.2.5**	Solid-gas synthesis of **[1-(ethene)$_2$][S-BAr$^F_4$]**	S12
	**S.2.6**	Synthesis of **[1-(ethene)$_2$][S-BAr$^F_4$]** in pentane suspension	S14
	**S.2.7**	Ethene coupling to 2-butene catalysed by **[1-NBA][S-BAr$^F_4$]** in pentane suspension	S15
	**S.2.8**	1-butene isomerisation catalysed by **[1-NBA][S-BAr$^F_4$]** in pentane suspension	S17
**S.3**	**CRYSTALLOGRAPHIC AND REFINEMENT DATA**		S18
	**S.3.1**	Single-crystal X-ray diffraction methods	S18
	**S.3.2**	MicroED methods	S18
	**S.3.3**	**[1-(NBD)][S-BAr$^F_4$]**	S20
	**S.3.4**	**[1-(NBA)][S-BAr$^F_4$]**	S21
	**S.3.5**	**[1-(ethene)$_2$][S-BAr$^F_4$]**	S24
**S.4**	**COMPUTATIONAL METHODS**		S25
	**S.4.1**	QTAIM study of **[1-NBA]$^+$**	S27
	**S.4.2**	Non-covalent interaction (NCI) study of the **[1-NBA][S-BAr$^F_4$]** ion-pair	S28
	**S.4.3**	Natural bond orbital analysis of the **[1-NBA]$^+$** cation	S29
	**S.4.4**	CrystalExplorer analysis of **[1-NBA][S-BAr$^F_4$]** and **[1-NBA][BAr$^F_4$]**	S30
	**S.4.5**	Computed cartesian coordinates (Å) and energy for **[1-NBA][S-BAr$^F_4$]**	S33
**S.5**	**REFERENCES**		S44

S.1 EXPERIMENTAL DETAILS

All manipulations (unless stated otherwise) were performed under an argon atmosphere, using standard Schlenk techniques on a dual vacuum/argon manifold or by using an argon filled glovebox (MBraun). Glassware was flame dried under vacuum prior to use. Pentane and dichloromethane (CH_2Cl_2) were dried using an Innovative Technology Pure-Solv™ (PS-400-3) solvent purification system and degassed by freeze-pump-thaw cycles. Deuterated solvents were dried using an appropriate drying agent: dichloromethane-d$_2$ (CD_2Cl_2) with CaH_2; acetonitrile-d$_3$ (MeCN-d$_3$) with 3 Å molecular sieves. After drying, these solvents were degassed by freeze-pump-thaw cycles and then stored over 3 Å molecular sieves. Hydrogen (H_2) and deuterium (D_2) gases were purchased in lecture bottles from Sigma-Aldrich and used as received. $[Rh(Cy_2P(CH_2)_2PCy_2)Cl]_2$ was prepared by a previously reported method.[1] All other chemicals were purchased from commercial vendors and used as received.

Solution NMR data were collected on either a Bruker AVIIIHD 500 MHz or 600 MHz spectrometer at 298 K unless otherwise started. Residual protio solvent resonances were used as a reference for ^{1}H NMR spectra.[2] ^{31}P{^{1}H} NMR spectra were referenced externally to 85 % H_3PO_4 (D_2O). All chemical shifts (δ) are quoted in ppm and coupling constants in Hz.

Solid state NMR (SSNMR) samples were prepared by packing powdered microcrystalline samples into a 4 mm zirconia solid state rotor inside an argon filled glove box. SSNMR spectra were obtained on a Bruker AVIIIHD 400 spectrometer, with a magic-angle spinning (MAS) rate of 10 kHz, referenced externally to triphenylphosphine (^{31}P: δ = –9.3) or adamantane (^{13}C{^{1}H}: upfield methine resonance, δ 29.5).[3]

Thermogravimetric analysis (TGA) and Differential Scanning Calorimetry (DSC) measurements were performed in a thermal analyser (Netzsch STA 449 F5 Jupiter®) using an alumina crucible. The samples were heated up to 1000 °C at a ramp rate of 10 °C min^{-1} under an atmosphere of He flowing at 20 mL min^{-1}.

The powder X-ray crystallography was performed on a Panalytical Aeris X-ray diffractometer equipped with a 600 W copper source and a PIXcel1D-Medipix3 detector. The instrument was operated in transmission mode with the sample in a 0.6mm OD borosilicate capillary.

Elemental microanalyses were carried out by Dr Graeme McAllister at the University of York using an Exeter Analytical CE-440 analyser.

S.2 SYNTHETIC PROCEDURES

S.2.1 Synthesis of [1-NBD][S-BAr$^F_4$]

Under an Ar pressure (1.2 bar), a solution of $[Rh(Cy_2P(CH_2)_2PCy_2)Cl]_2$ (70 mg, 0.062 mmol) in CH_2Cl_2 (5 mL) was quickly added to an ampoule containing a solution of $[Bu_4N][S\text{-}BArF_4]$ (188 mg, 0.120 mmol) and NBD (50 µL, 0.492 mmol) dissolved in refluxing CH_2Cl_2 (5 mL, 40 °C, 1.2 bar). After mixing, the solution was then filtered into a clean ampoule using a cannular adapted with a PTFE-wrapped glass microfiber filter pad. Bright orange microcrystals of **[1-NBD][S-BAr$^F_4$]** began forming almost immediately and the mixture was left at room temperature for a further 15 h. The supernatant was then decanted from the crystals, which were washed with additional CH_2Cl_2 (3 x 5 mL) and pentane (3 x 5 mL), then dried *in vacuo* at 10^{-3} mbar for at least 15 h. Larger crystals that were suitable for XRD measurements were obtained by keeping the above filtrate at 40 °C during crystal growth. Yield: 197 mg (0.101 mmol, 85 %).

Elemental analysis found (calculated): C 35.34 (35.19), H 3.58 (3.52).

$^{31}P\{^1H\}$ SSNMR (162 MHz, 10 kHz spin rate, 290 K): δ 75.67 (d, $J_{Rh\text{-}P} \approx$ 130 Hz).

$^{13}C\{^1H\}$ SSNMR (101 MHz, 10 kHz spin rate, 290 K): δ 162.07-164.40 (br m, S-BAr$^F_4$), 155.40 (s, S-BAr$^F_4$), 154.85 (s, S-BAr$^F_4$), 135.62 (s, S-BAr$^F_4$), 133.77 (s, S-BAr$^F_4$), 121.07 (s, S-BAr$^F_4$), 91.16 (s, NBD, alkene-CH), 84.09 (s, NBD, alkene-CH), 69.48 (s, NBD, CH2 bridge), 56.28 (s, NBD, bridgehead), 39.17, 36.23, 32.67, 29.65, 28.81, 27.54, 27.01, 26.56, 23.55 (multiple overlapping aliphatic resonances).

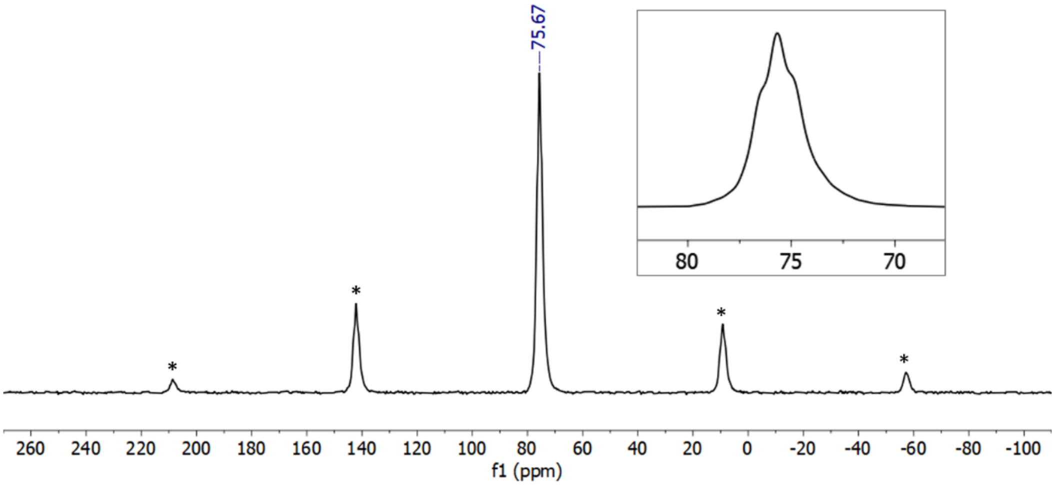

Figure S1. $^{31}P\{^1H\}$ SSNMR spectrum of **[1-NBD][S-BAr$^F_4$]**. Recorded at 290 K and a MAS rate of 10 KHz; * denotes spinning sidebands.

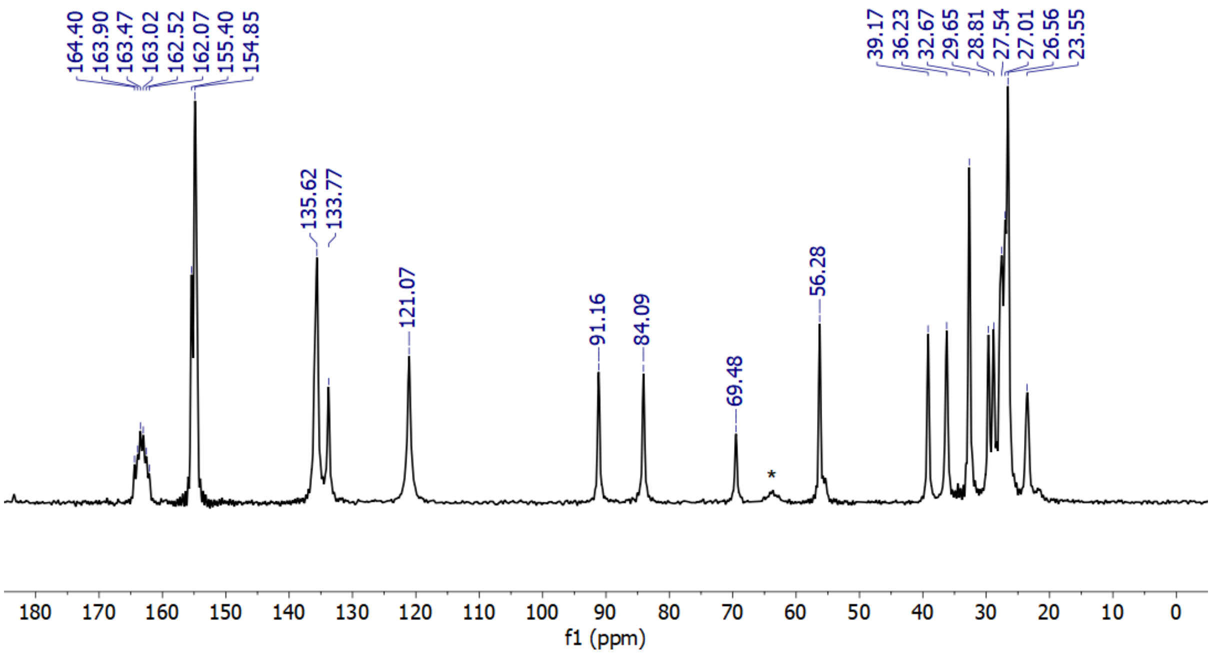

Figure S2. $^{13}C\{^1H\}$ SSNMR spectrum of **[1-NBD][S-BAr$^F_4$]**. Recorded at 290 K and a MAS rate of 10 KHz; * denotes a spinning sideband.

S.2.2 Synthesis of [1-NBA][S-BAr$^F_4$]

A microcrystalline sample of **[1-NBD][S-BAr$^F_4$]** (78 mg, 0.04 mmol) was hydrogenated with H_2 (20 PSI) for 30 min, resulting in quantitative conversion to reddish orange **[1-NBA][S-BAr$^F_4$]**.

Elemental analysis found (calculated): C 34.71 (35.12), H 3.90 (3.72).

$^{31}P\{^1H\}$ SSNMR (162 MHz, 10 kHz spin rate, 293 K): δ 110.05 (two overlapping d, $J_{Rh\text{-}P}$ = 196 Hz). 185 K: δ 110.98 (d, $J_{Rh\text{-}P}$ = 194 Hz), 107.42 (d, $J_{Rh\text{-}P}$ = 201 Hz).

$^{13}C\{^1H\}$ SSNMR (101 MHz, 10 kHz spin rate, 293 K): δ 163.27 (br m, S-BAr$^F_4$), 155.37 (s, S-BAr$^F_4$), 154.78 (s, S-BAr$^F_4$), 135.53 (s, S-BAr$^F_4$), 134.00 (s, S-BAr$^F_4$), 121.30 (br s, S-BAr$^F_4$), 44.51, 40.65, 37.34, 33.14, 32.23, 31.15, 27.09, 21.62 (multiple overlapping aliphatic resonances). 185 K: δ 162.56 (br m, S-BAr$^F_4$), 154.92 (s, S-BAr$^F_4$), 154.11 (s, S-BAr$^F_4$), 134.67 (br m, S-BAr$^F_4$), 121.29 (s, S-BAr$^F_4$), 120.49 (s, S-BAr$^F_4$), 44.86, 41.05, 39.81, 37.28, 36.13, 33.34, 28.97, 28.09, 27.13, 26.28, 21.88, 20.98 (multiple overlapping aliphatic resonances).

Powder XRD (298 K): a = 19.597(4) Å, b = 19.302(3) Å, c = 20.467(3) Å, α = 90°, β = 91.792(3)°, γ = 90°, volume = 7738 Å^3.

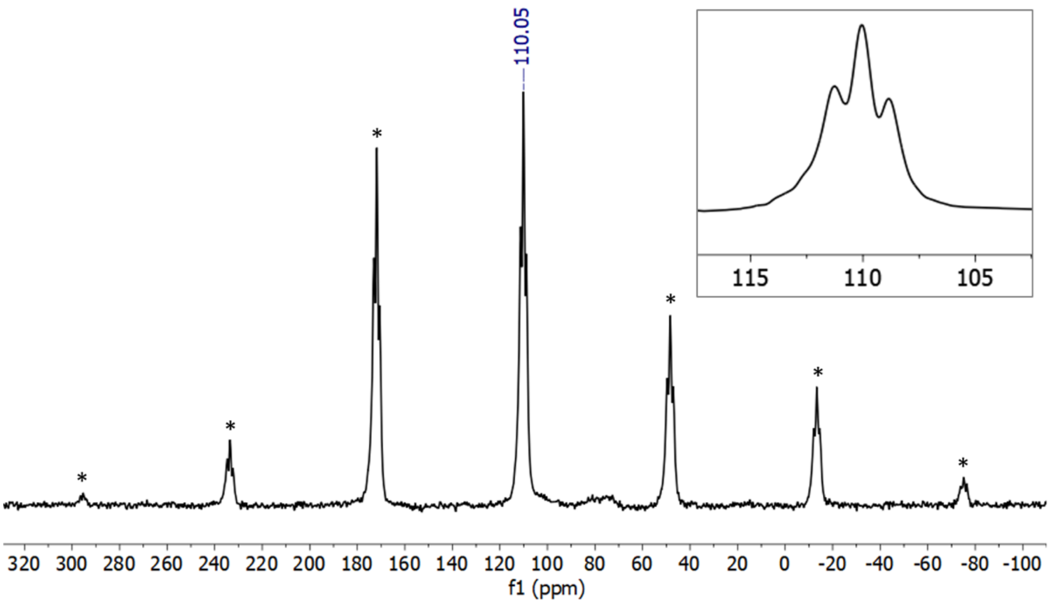

Figure S3. $^{31}P\{^1H\}$ SSNMR spectrum of **[1-NBA][S-BAr$^F_4$]**. Recorded at 293 K and a MAS rate of 10 KHz; * denotes spinning sidebands.

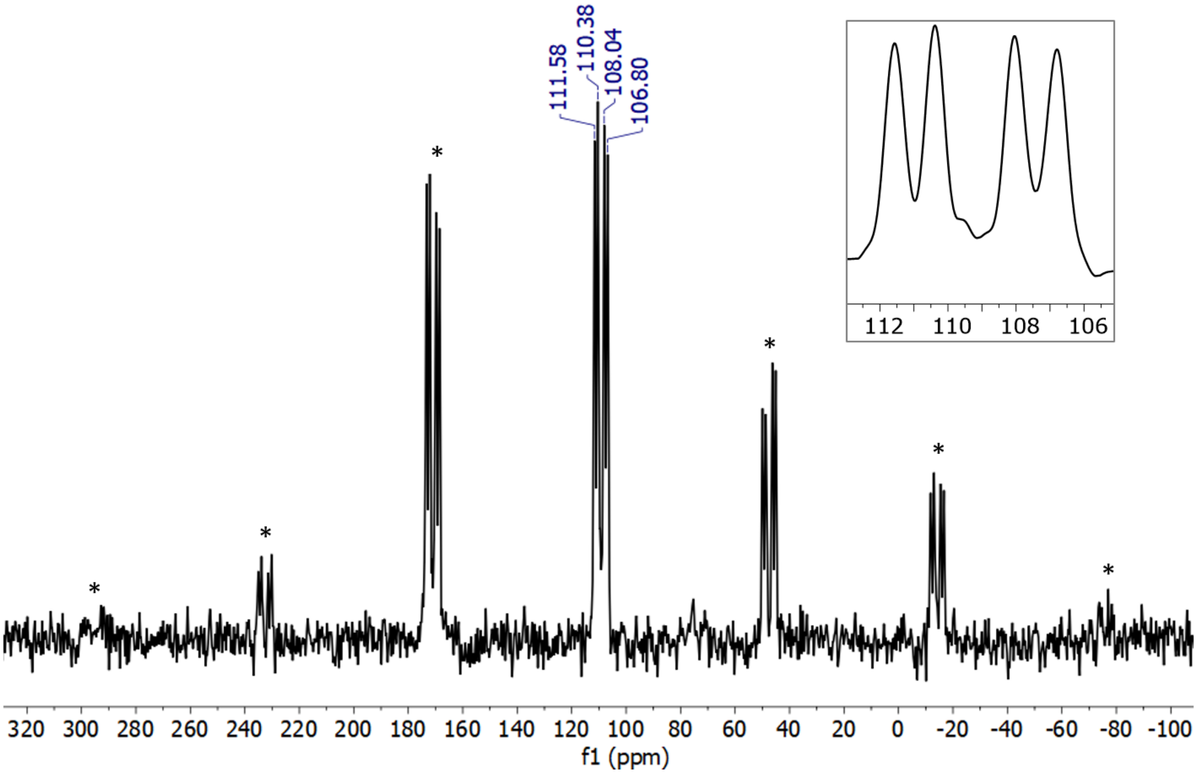

Figure S4. $^{31}P\{^1H\}$ SSNMR spectrum of **[1-NBA][S-BAr$^F_4$]**. Recorded at 185 K and a MAS rate of 10 KHz; * denotes spinning sidebands.

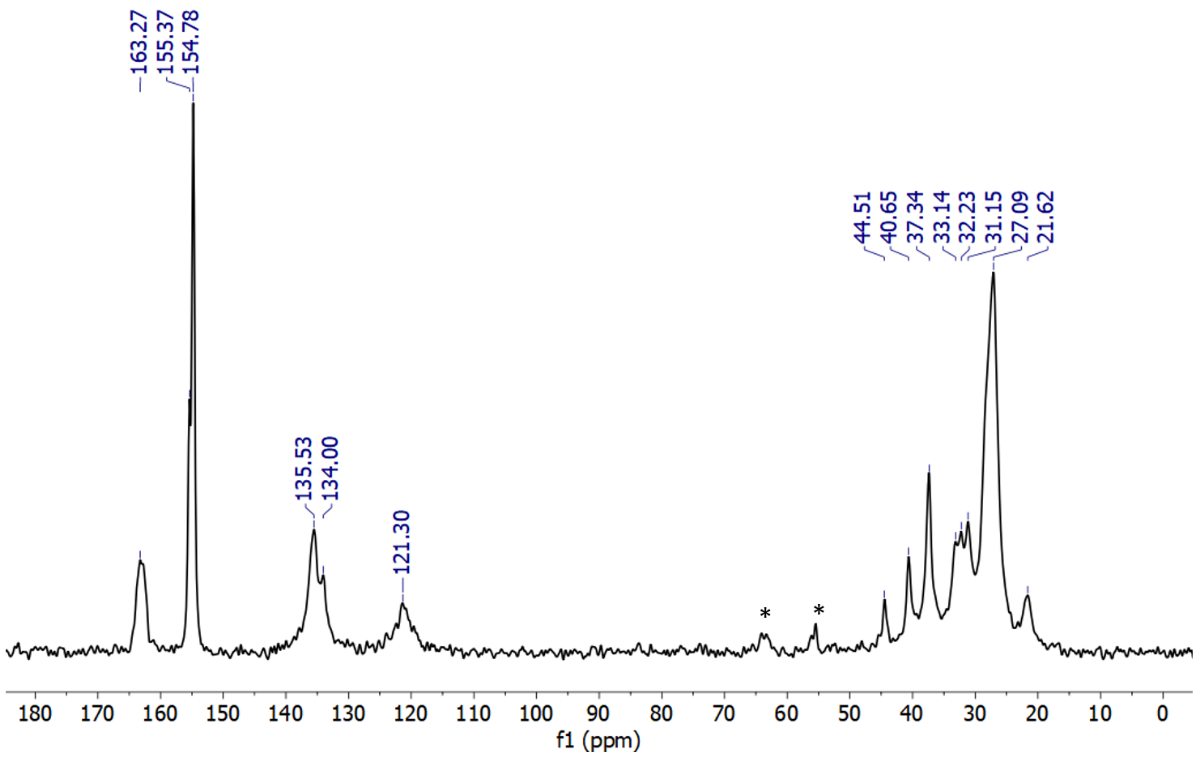

Figure S5. $^{13}C\{^{1}H\}$ SSNMR spectrum of **[1-NBA][S-BAr$^{F}_{4}$]**. Recorded at 293 K and a MAS rate of 10 KHz; * denotes spinning sidebands.

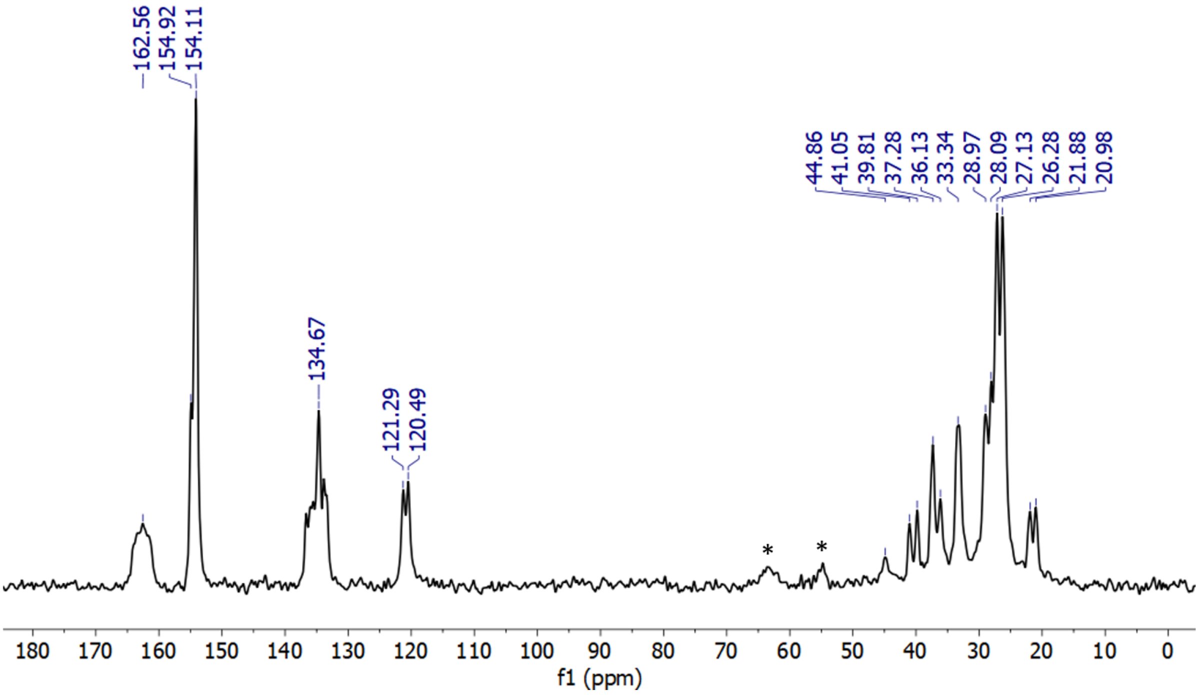

Figure S6. $^{13}C\{^{1}H\}$ SSNMR spectrum of **[1-NBA][S-BAr$^{F}_{4}$]**. Recorded at 185 K and a MAS rate of 10 KHz; * denotes spinning sidebands.

S.2.3 Synthesis of endo-d$_4$-[1-NBA][S-BAr$^F_4$], exo-d$_4$-[1-NBA][S-BAr$^F_4$], and d$_8$-[1-NBA][S-BAr$^F_4$]

Microcrystalline samples of **[1-NBD][S-BAr$^F_4$]** (9.7 mg, 0.005 mmol) were hydrogenated with either H_2 or D_2 (20 PSI) in one or two stages as follows. **[1-NBA][S-BAr$^F_4$]** or **endo-d$_4$-[1-NBA][S-BAr$^F_4$]** are formed with one, 3 min hydrogenation with H_2 or D_2, respectively (Figure S7). These species undergo H/D exchange with additional D_2 exposure over 20 hr to form **exo-d$_4$-[1-NBA][S-BAr$^F_4$]** and **d$_8$-[1-NBA][S-BAr$^F_4$]**, respectively.

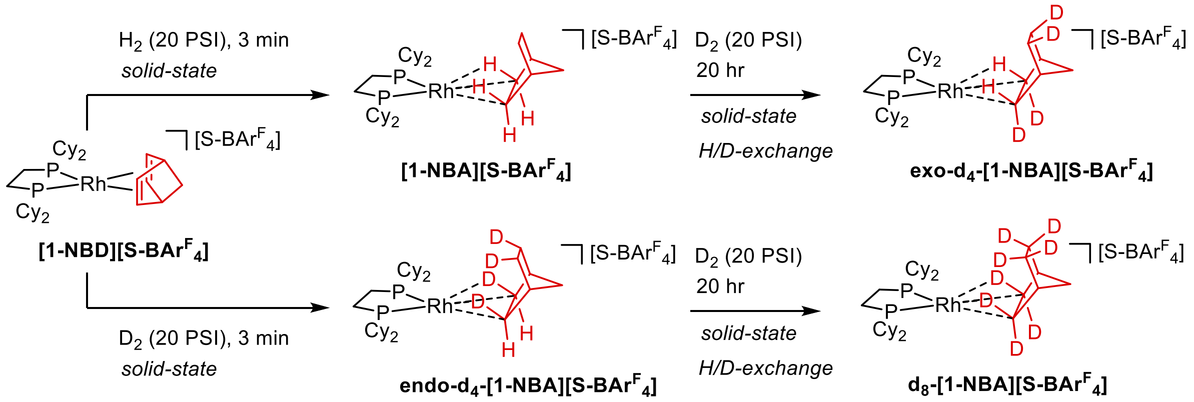

Figure S7. Syntheses of of **d$_x$-[1-NBA][S-BAr$^F_4$]** (x=0, 4, 8).

<u>Volatile trapping of displaced d$_x$-NBA</u>

Microcrystalline samples (0.005 mmol) of **d$_x$-[1-NBA][S-BAr$^F_4$]** were treated with CD_2Cl_2 (0.5 mL) and MeCN-d$_3$ (10.6 μL, 40 eq.), immediately resulting in a yellow solution, from which a yellow solid precipitated. The volatiles containing liberated d$_x$-NBA were vacuum transferred to an empty NMR tube, then sealed under an Ar atmosphere for subsequent NMR analysis.

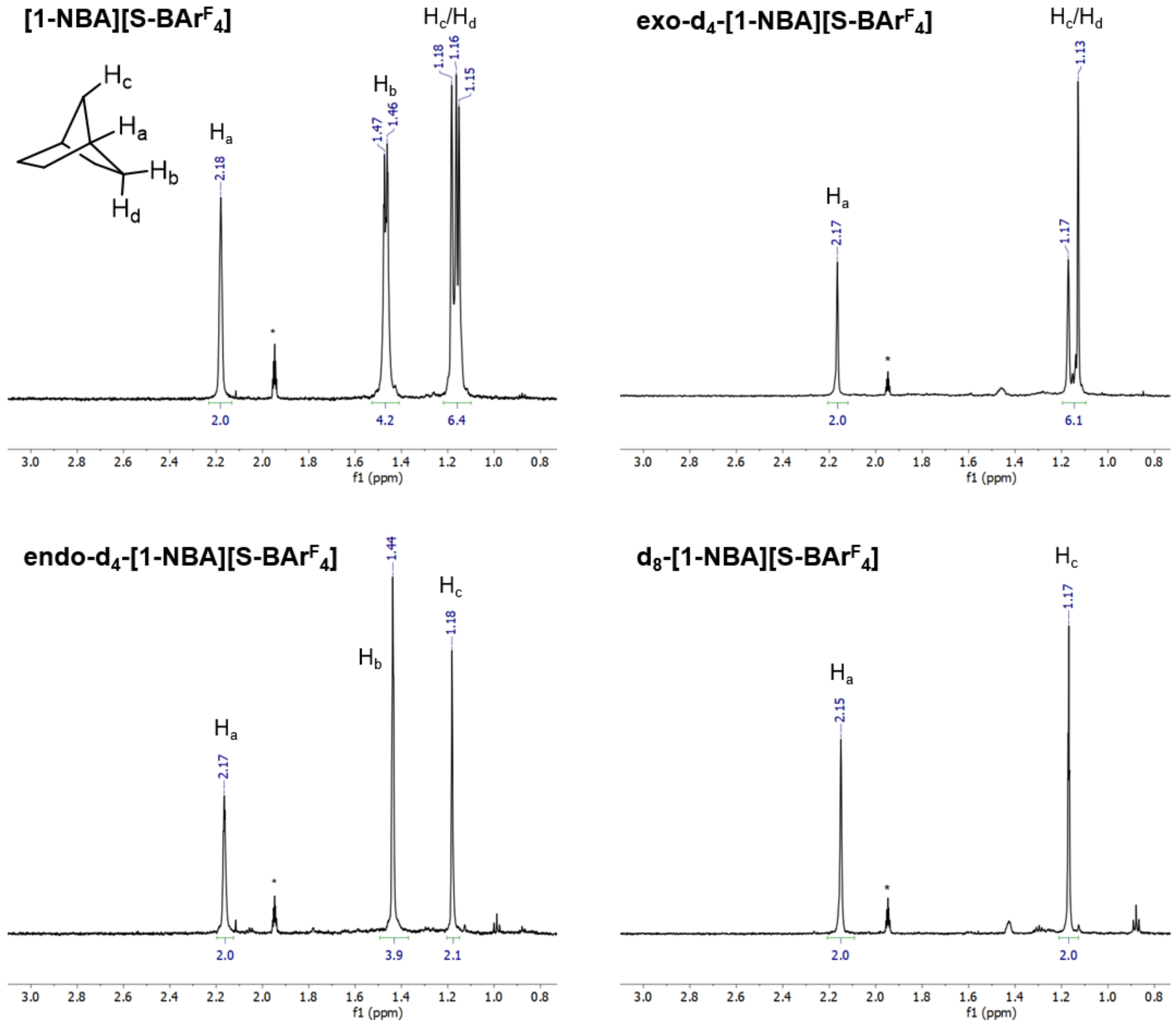

Figure S8. ^{1}H NMR spectra of **d_x-NBA** liberated from **d_x-[1-NBA][S-BAr^F$_4$]** by dissolution in MeCN-d_3 (10.6 μL) and CD_2Cl_2 (0.5 mL) followed by vacuum distillation.

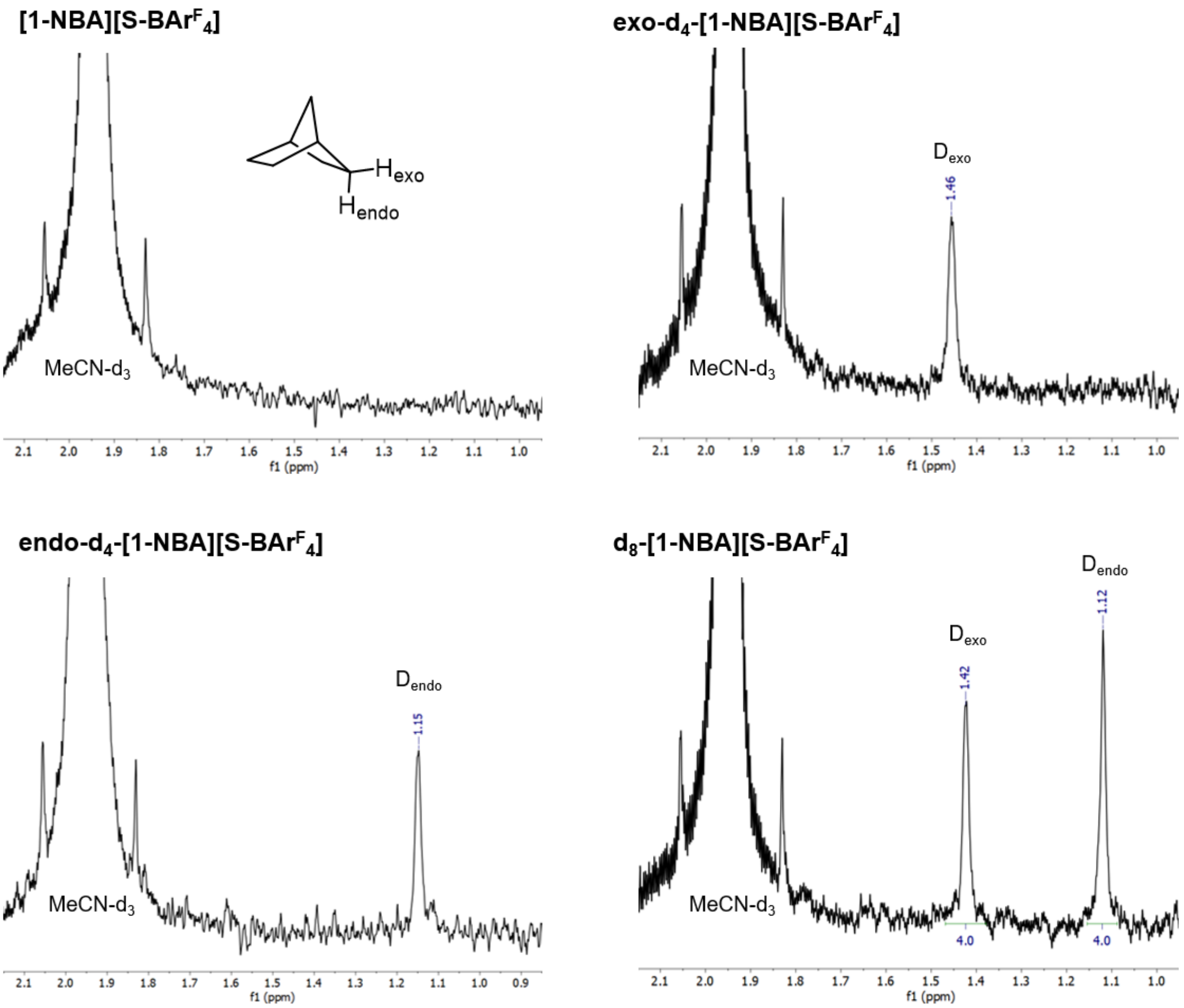

Figure S9. $^{2}H\{^{1}H\}$ NMR spectra of **d_x-NBA** liberated from **d_x-[1-NBA][S-BAr$^F_4$]** by dissolution in MeCN-d_3 (10.6 μL) and CD_2Cl_2 (0.5 mL) followed by vacuum distillation.

S.2.4 Stability of [1-NBA][S-BAr$^F_4$] and [1-NBA][BAr$^F_4$] in pentane

Separately, ground samples of **[1-NBD][S-BAr$^F_4$]** (60 mg, 0.031 mmol) and **[1-NBD][BAr$^F_4$]** (60 mg, 0.041 mmol) were hydrogenated with H_2 (20 PSI) for 30 min to form **[1-NBA][S-BAr$^F_4$]** and **[1-NBA][BAr$^F_4$]**, respectively. Within an Ar glovebox, these were dissolved in Ar-saturated pentane (1 mL) and stirred for 2 hrs. **[1-NBA][S-BAr$^F_4$]** remained as a fine suspension throughout, whereas **[1-NBA][BAr$^F_4$]** formed an orange oil at the solid-liquid interface, which initially prevented stirring; after physically dislodging the stuck stirrer bar, the stirred mixture formed a fine suspension of pale yellow **1-BAr$^F_4$** within 2 hrs. After removing all volatiles in vacuo (0.01 mbar), the remaining solids were analysed by $^{31}P\{^{1}H\}$ SSNMR.

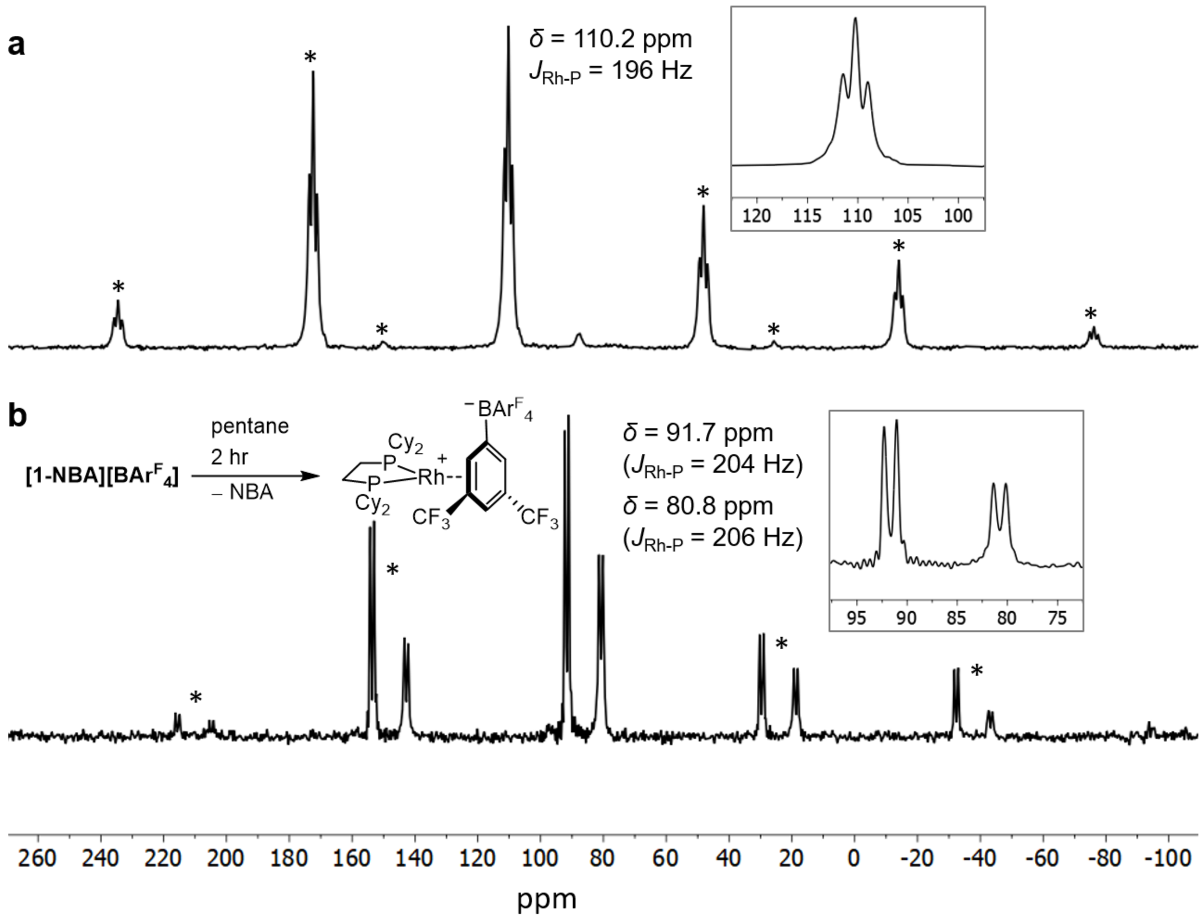

Figure S10. $^{31}P\{^{1}H\}$ SSNMR spectra of (a) **[1-NBA][S-BAr^{F}$_4$]** and (b) **[1-NBA][BAr^{F}$_4$]** after stirring in pentane for 2 hr. Recorded at 290 K and a MAS rate of 10 KHz; * denotes spinning sidebands.

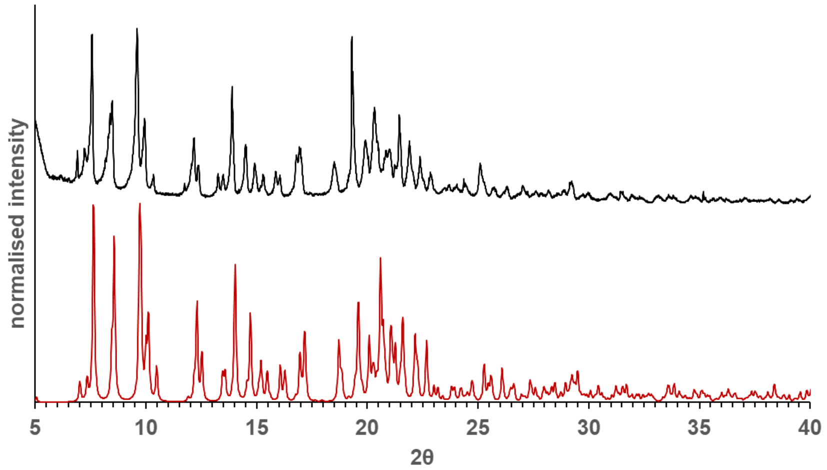

Figure S11. Powder diffraction pattern of **1-BAr^{F}$_4$** measured at 298 K (top, black) versus simulated diffraction pattern (bottom, red) generated using single crystal data for this complex measured at 150 K (CCDC no. 1022727).[2]

S.2.5 Solid-gas synthesis of [1-(ethene)$_2$][S-BAr$^F_4$]

In a ca 15 cm^3 Rotaflo® ampoule, a microcrystalline sample of **[1-NBD][S-BAr$^F_4$]** (78 mg, 0.04 mmol) was hydrogenated with H_2 (1.4 bar) for 30 min to form reddish orange **[1-NBA][S-BAr$^F_4$]**. The H_2 headspace was removed in vacuo (0.01 mbar) then replaced with ethene (4 bar) and left sealed for 24 hr to form orange **[1-(ethene)$_2$][S-BAr$^F_4$]**. The $^{31}P\{^1H\}$ SSNMR shows impurities that likely correspond to the butene and butadiene complexes, and are similar to those observed previously in the solid-gas synthesis of **[1-(ethene)$_2$][BAr$^F_4$]**, reported previously.[3]

$^{31}P\{^1H\}$ SSNMR (162 MHz, 10 kHz spin rate, 298 K): *δ* 75.57 (br s, fwhm ≈ 530 Hz).

$^{13}C\{^1H\}$ SSNMR (101 MHz, 10 kHz spin rate, 298 K): *δ* 163.48 (br s, S-BAr$^F_4$), 155.35 (s, S-BAr$^F_4$), 135.60 (s, S-BAr$^F_4$), 121.12 (s, S-BAr$^F_4$), 84.36 (br s, C_2H_4), 47.53 (s, S-BAr$^F_4$), 40.75, 39.80, 37.87, 32.37, 31.21, 28.10, 27.07, 23.15 (multiple overlapping aliphatic resonances). 198 K: 163.06 (br s, S-BAr$^F_4$), 154.81 (s, S-BAr$^F_4$), 135.45 (s, S-BAr$^F_4$), 121.01 (s, S-BAr$^F_4$), 83.86 (br s, C_2H_4), 47.20 (s, S-BAr$^F_4$), 39.59, 37.84, 37.17, 31.09, 27.77, 27.02, 19.22, 16.71 (multiple overlapping aliphatic resonances).

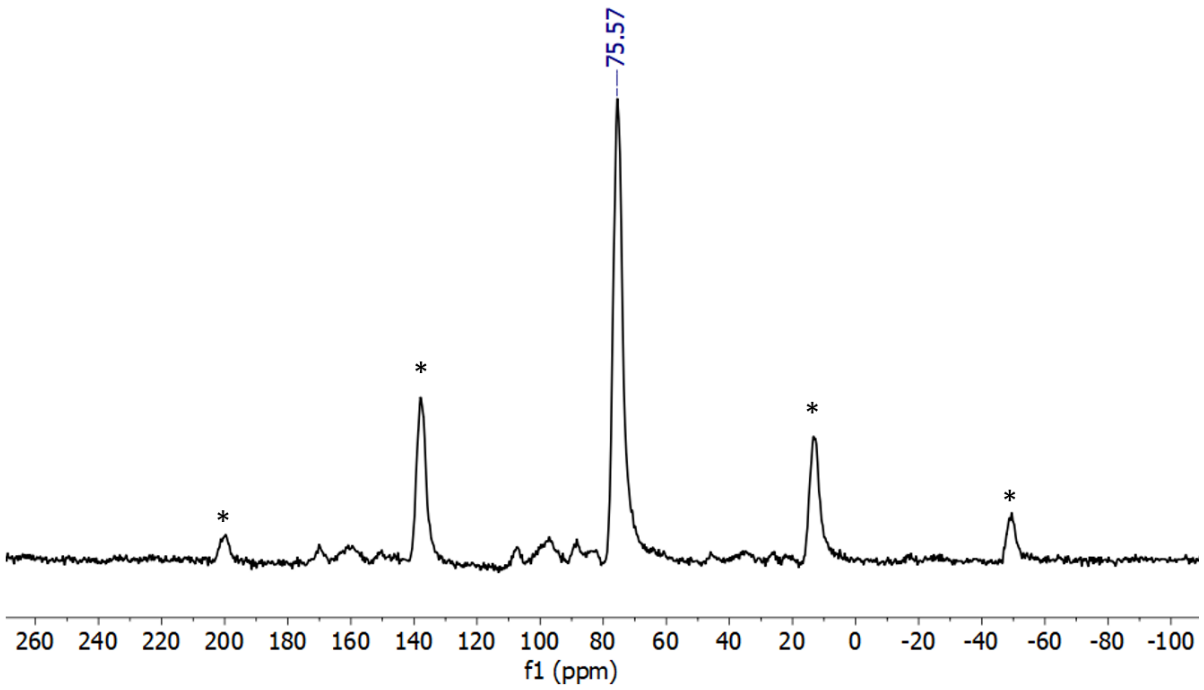

Figure S12. $^{31}P\{^1H\}$ SSNMR spectrum of **[1-(ethene)$_2$][S-BAr$^F_4$]** synthesised by the solid-gas method. Recorded at 298 K and a MAS rate of 10 KHz; * denotes spinning sidebands for the major resonance at 75.57 ppm.

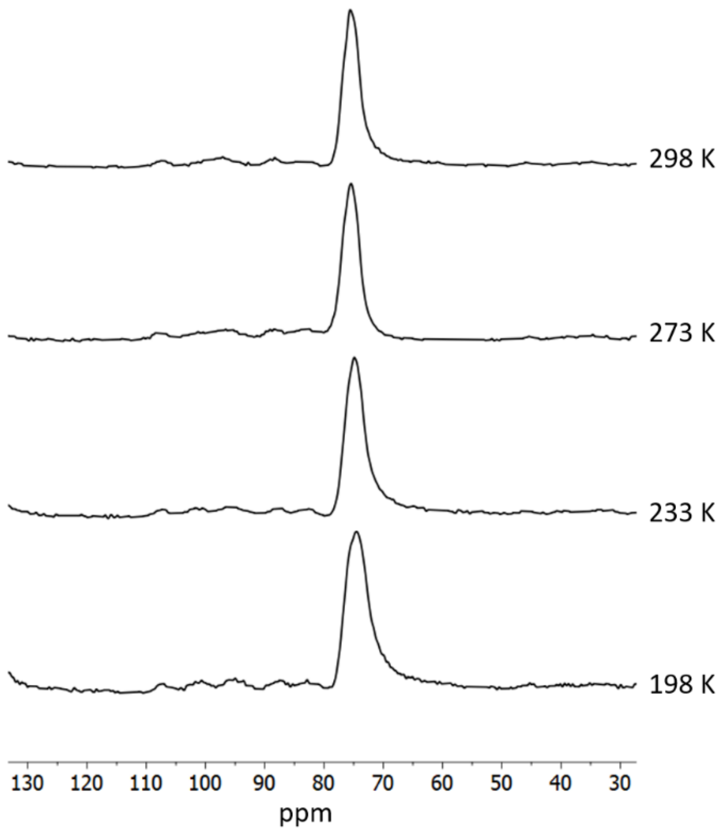

Figure S13. $^{31}P\{^{1}H\}$ VT-SSNMR spectra of **[1-(ethene)$_2$][S-BAr$^F{}_4$]** synthesised by the solid-gas method. Recorded at 298-198 K and a MAS rate of 10 KHz.

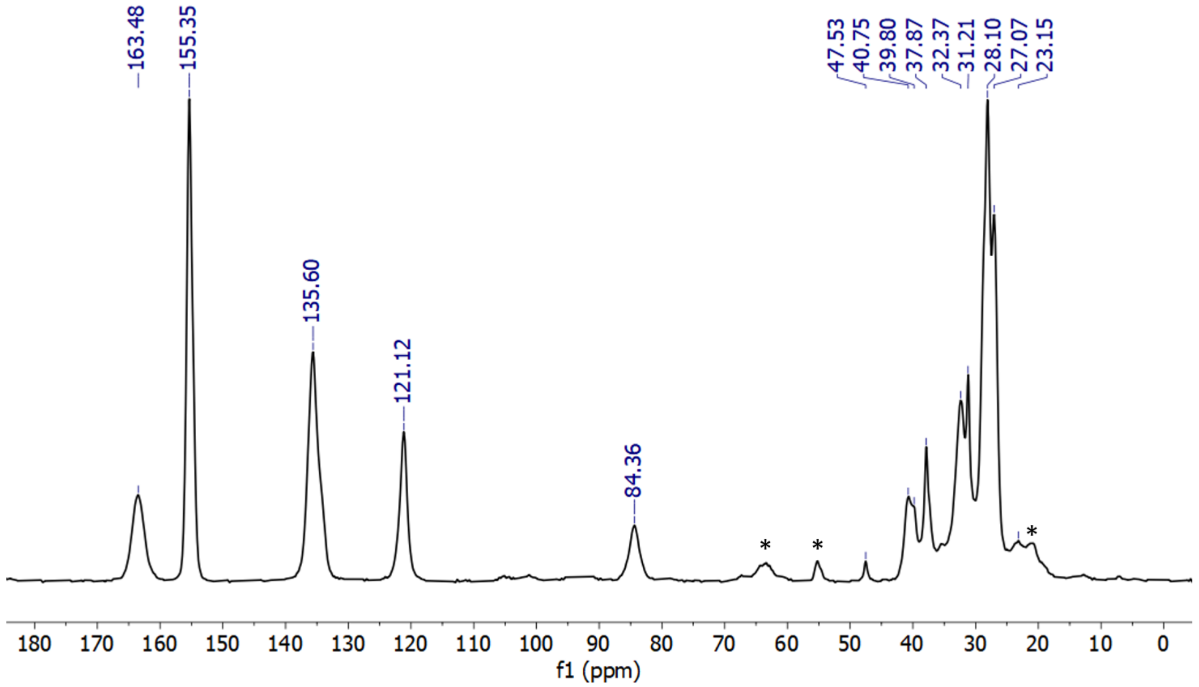

Figure S14. $^{13}C\{^{1}H\}$ SSNMR spectrum of **[1-NBA][S-BAr$^F{}_4$]** synthesised by the solid-gas method. Recorded at 298 K and a MAS rate of 10 KHz; * denotes spinning sidebands.

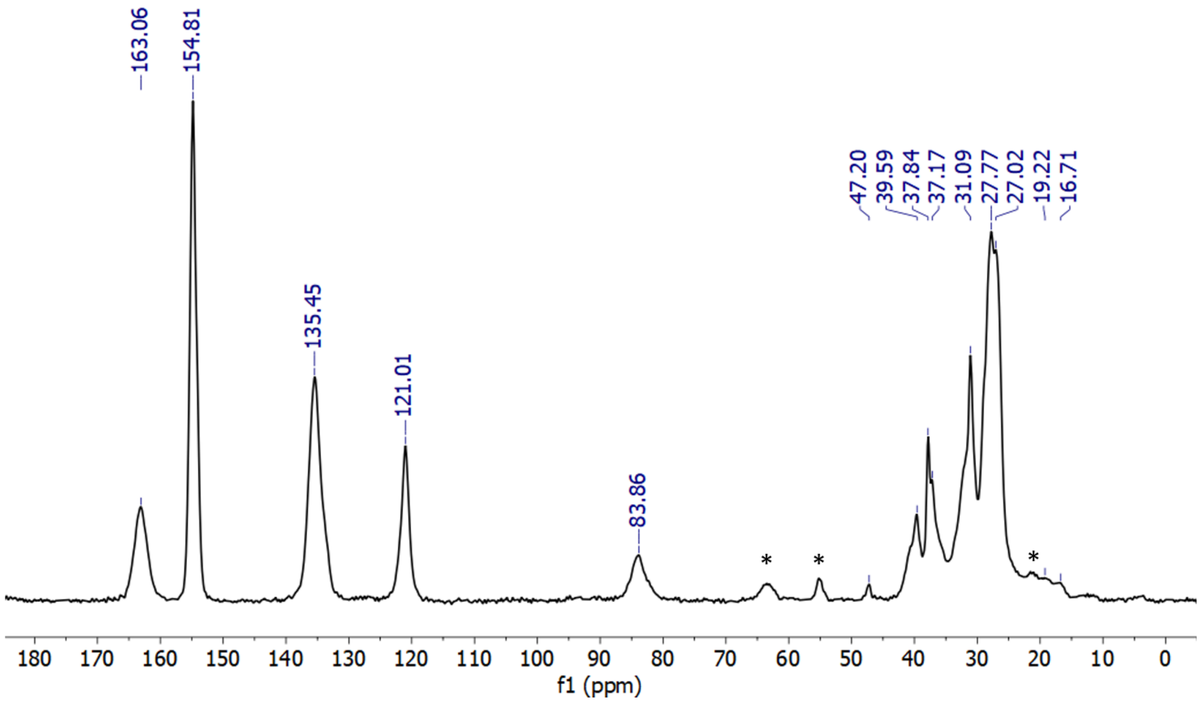

Figure S15. $^{13}C\{^1H\}$ SSNMR spectrum of **[1-NBA][S-BAr$^F_4$]** synthesised by the solid-gas method. Recorded at 198 K and a MAS rate of 10 KHz; * denotes spinning sidebands.

S.2.6 Synthesis of [1-(ethene)$_2$][S-BAr$^F_4$] in pentane suspension

A microcrystalline sample of **[1-NBD][S-BAr$^F_4$]** (60 mg, 0.031 mmol) was suspended in pentane (1 mL) in a 10 cm^3 ampoule with a J. Young valve and fitted with a magnetic stirrer bar. After three freeze-pump-thaw degassing cycles, the ampoule was placed under a H_2 (20 PSI) atmosphere and the mixture was stirred for 30 min, forming a suspension of reddish orange **[1-NBA][S-BAr$^F_4$]**. After three freeze-pump-thaw degassing cycles, the ampoule was recharged and sealed under an atmosphere of ethene (20 PSI). After stirring for 20 hr, a 0.5 mL aliquot was taken (see below), then all volatiles were removed in vacuo (0.01 mbar, 30 min) to isolate **[1-(ethene)$_2$][S-BAr$^F_4$]** as an orange solid.

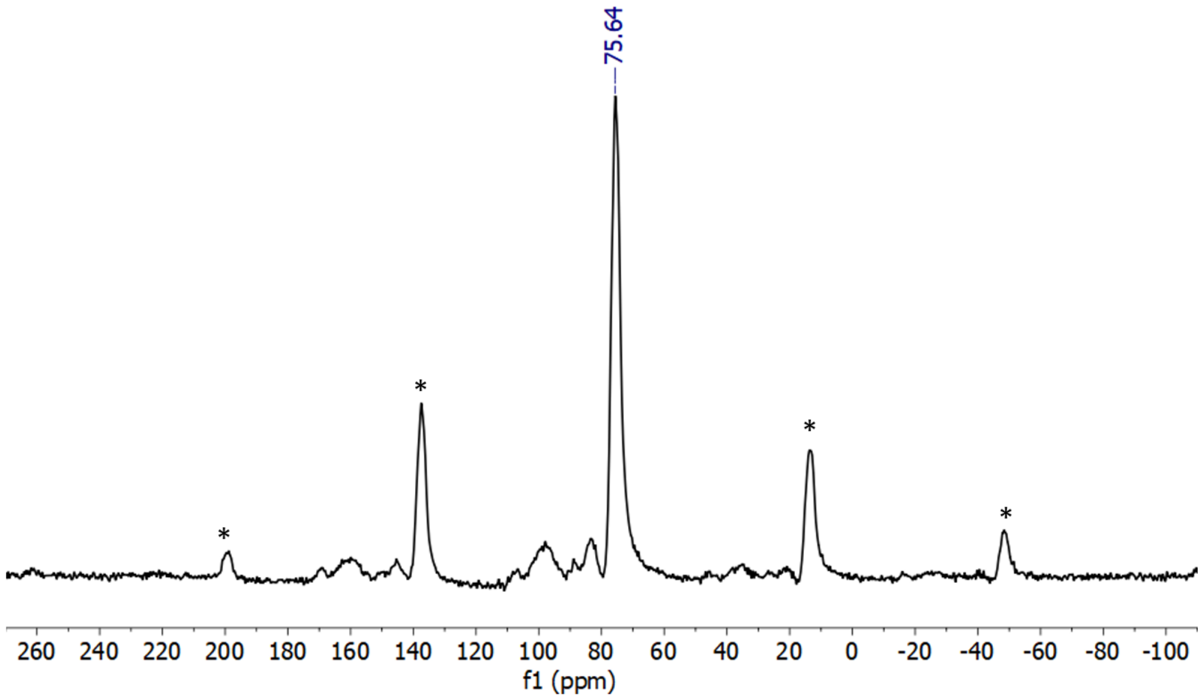

Figure S16. $^{31}P\{^1H\}$ SSNMR spectrum of **[1-(ethene)$_2$][S-BAr^F$_4$]** synthesised by the pentane slurry method. Recorded at 298 K and a MAS rate of 10 KHz; * denotes spinning sidebands for the major resonance at 75.64 ppm.

S.2.7 Ethene coupling to 2-butene catalysed by [1-NBA][S-BAr^F$_4$] in pentane suspension

Experiment 1: A microcrystalline sample of **[1-NBD][S-BAr^F$_4$]** (15 mg, 0.0077 mmol) was weighed into a 10 cm^3 J. Young ampoule fitted with a magnetic stirrer bar. The sample was hydrogenated with H_2 (20 PSI, 10 min) to form **[1-NBA][S-BAr^F$_4$]**, then suspended in pentane (1 mL). After three freeze-pump-thaw degassing cycles, the ampoule was charged and sealed under an atmosphere of ethene (20 PSI, ~9 cm^3, ~66 eq. per Rh) and stirred at 500 rpm. After 20 hr, an internal reference, adamantane (15 mg, 0.11 mmol), was added to the mixture, which was then filtered through a 0.2 μm pore PTFE syringe filter into a J. Young NMR tube. ^{1}H NMR analysis of this pentane solution, integrated relative to the adamantane reference, revealed liberated NBA, 2-butenes, 1-butene and unreacted ethene (Table 1). The ampoule containing the remaining solids was subsequently recharged with pentane (1 mL) and ethene (20 PSI) as before. The mixture was stirred for a further 20 hr, then quantified once more by ^{1}H NMR, relative to additional adamantane. To examine whether any trace, unobservable but active, soluble species were present, the filtered solution taken after the first 20 hr was recharged with ethene, stirred for 20 hr, then reanalysed by ^{1}H NMR: no additional 2-butenes or 1-butene had formed over this time. The analogous reaction with **[1-NBA][BAr^F$_4$]** yielded less than 0.1 equivalents of 2-butene per Rh.

Table 1. [1-NBA][S-BAr$^{F}_{4}$] catalysed ethene coupling in pentane suspension: quantification by ^{1}H NMR.

	1st cycle (20 hr)	2nd cycle (+20 hr)	Total (40 hr)
**NBA** (eq. per Rh)	0.93	0.07	1.0
**2-butenes** (eq. per Rh)	6.9	7.4	14.3
**1-butene** (eq. per Rh)	0.21	0.21	0.42

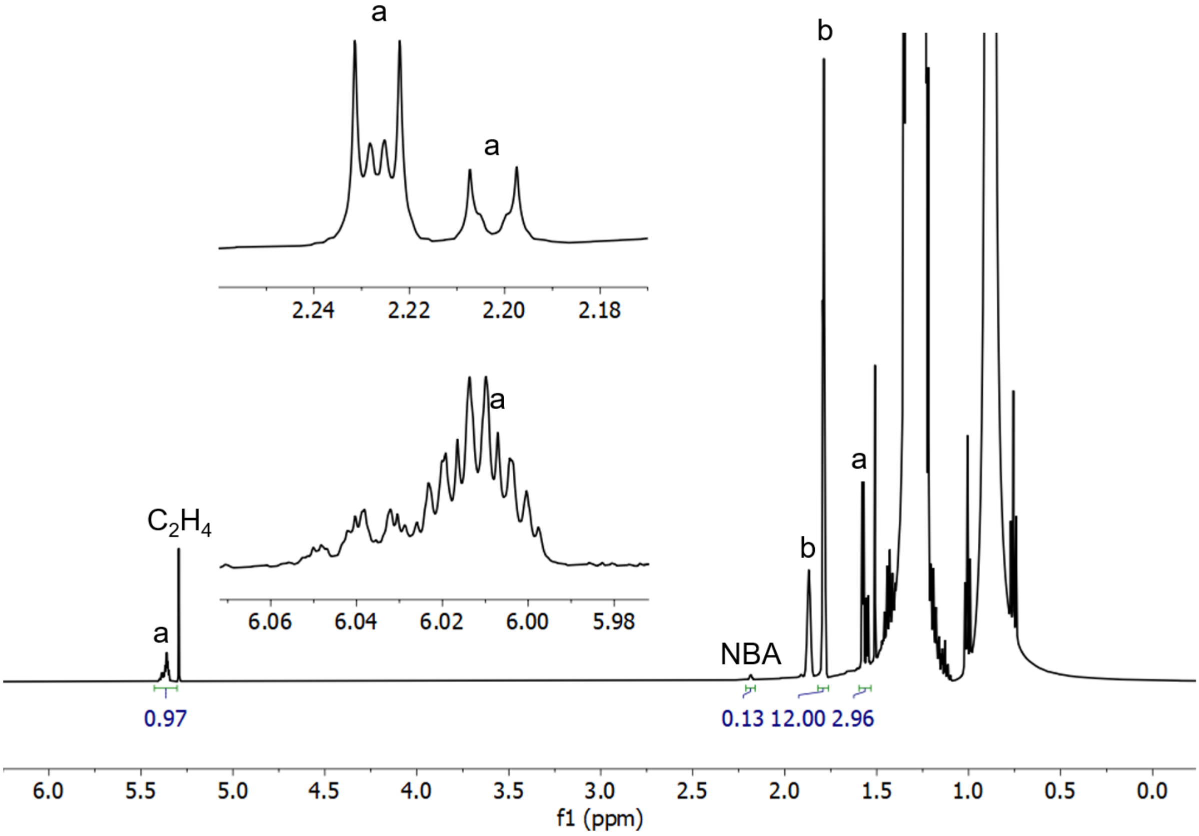

Figure S17. Exemplar ^{1}H NMR spectrum of the reaction of ethene with a pentane suspension of **[1-NBA][S-BAr$^{F}_{4}$]**, extracted after the first 20 hr cycle; a = 2-butenes, b = adamantane reference.

Experiment 2: The above synthesis of **[1-(ethene)$_2$][S-BAr$^{F}_{4}$]** from **[1-NBD][S-BAr$^{F}_{4}$]** (60 mg, 0.031 mmol) via **[1-NBA][S-BAr$^{F}_{4}$]** in a pentane suspension was conducted primarily to assess the solid reaction product by SS NMR analysis, however, the mixture was also assessed for 2-butenes and 1-butene by ^{1}H NMR analysis of the pentane supernatant, using the quantitatively displaced NBA (1 eq. per Rh) as an internal reference. After 20 hr, a 0.5 mL aliquot was removed prior to isolation of the solids for SS NMR characterisation (Table 2). The solids – predominantly **[1-(ethene)$_2$][S-BAr$^{F}_{4}$]** – were resuspended in pentane (1 mL), after

which the ampoule headspace was recharged with ethene (20 PSI). After stirring for a further 20 hr, a second aliquot was taken (total: 2 cycles, 40 hr).

Table 2. [1-NBA][S-BAr$^F_4$] (formed in situ) catalysed ethene coupling in pentane suspension: quantification by ^{1}H NMR.

	Aliquot 1 (20 hr)	Aliquot 2 (+20 hr)	Total (40 hr)
**2-butenes** (eq. per Rh)	7.5	7.2	14.7
**1-butene** (eq. per Rh)	0.2	0.2	0.4

S.2.8 1-butene isomerisation catalysed by [1-NBA][S-BAr$^F_4$] in pentane suspension

The method described in Experiment 1 above was repeated using a 15 cm^3 J. Young ampoule and 1-butene (20 PSI, 14 cm^3, ~100 eq.) instead of ethene. An aliquot taken after 20 hr was analysed by quantitative ^{1}H NMR (versus an internal adamantane reference), which found 2-butenes (~98 eq. per Rh) and 1-butene (~2 eq. per Rh).

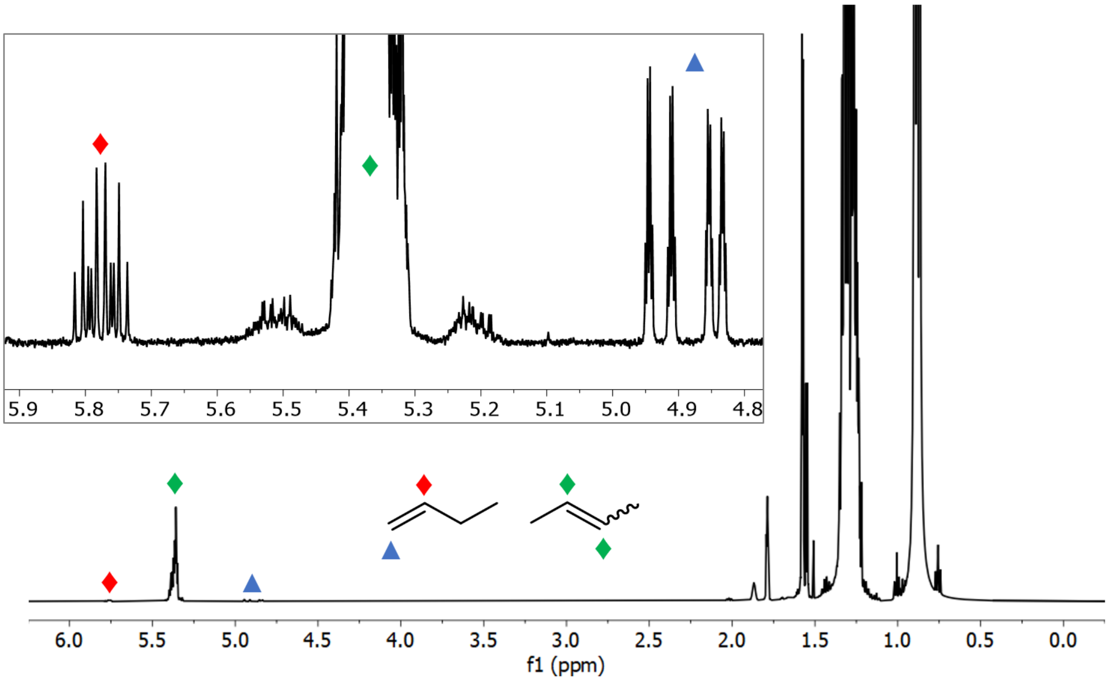

Figure S18. ^{1}H NMR spectrum of the reaction of 1-butene with a pentane suspension of **[1-NBA][S-BAr$^F_4$]**, recorded after 20 hr.

S.3 CRYSTALLOGRAPHIC AND REFINEMENT DATA

Selected crystallographic data are summarized in the text and full details are given in the supplementary deposited CIF files. This data can be obtained free of charge from the Cambridge Crystallographic Data Centre via http://www.ccdc.cam.ac.uk/data_request/cif. Electron diffraction data are also available from Zenodo (doi:10.5281/zenodo.5760938).

S.3.1 Single-crystal X-ray diffraction methods

Single-crystal X-ray diffraction data for **[1-NBD][S-BAr$^F{}_4$]**, **[1-NBA][S-BAr$^F{}_4$]**, and **[1-(ethene)$_2$][S-BAr$^F{}_4$]** were collected on an Oxford Diffraction SuperNova diffractometer with Cu-*Kα* (λ = 1.54184 Å) radiation equipped with a nitrogen gas Oxford Instruments Cryojet cooler. Raw frame data was reduced using CrysAlisPro, solved using Superflip[4], and refined using full-matrix least squares refinement on all F^2 data using SHELXL-18[5] within the OLEX2 program.[6] All non-hydrogen atoms were refined anisotropically and hydrogen atoms were geometrically placed unless otherwise stated and allowed to ride on their parent atoms. Distances and angles were calculated using the full covariance matrix.

S.3.2 MicroED methods

Micro-crystalline **[1-NBD][S-BAr$^F{}_4$]** was finely ground and deposited onto Quantifoil Cu R1/4 grids that had been assembled into autogrid cartridges. These grids were then treated with H_2 (1.3 atm for 5 minutes) then placed into a grid box and transported to the microscope under an Ar atmosphere. The grids were then transferred to the cassette under a blanket of N_2 vapour and conductively cooled to liquid nitrogen temperature before loading into the TEM.

MicroED data were collected using a Thermo Fisher Glacios microscope operated at 200 kV and equipped with a Ceta-D camera. A low flux of of ~0.01 $e^{-}Å^{-2}s^{-1}$ was achieved using the following illumination conditions: gun lens 4, spot size 11, 30 μm C2 aperture. This resulted in an illuminated area of 4 μm. Crystals were isolated using a 40 μm selected area aperture (1.4 μm on the sample plane). Data were acquired using EPU-D with the following settings: 2x binning, a rotation speed of 0.5°/s and an exposure time of 2 s. For **[1-NBD][S-BAr$^F{}_4$]** datasets were collected from 28 crystals over a rotation range of 60-90° between minimum and maximum tilt angles of -45°/+55°. **[1-NBA][S-BAr$^F{}_4$]** crystals were highly radiation sensitive and it was only possible to collect 20-30° of data before visible loss of diffraction quality occurred. Over the course of this work 111 datasets were collected from this sample but the

highest quality data were recorded from 29 crystals across 2 duplicate grids from the same microscope session.

All data were processed using DIALS[7]. The images recorded on Ceta-D camera show mean negative background values at high resolution which hampers background modelling so a pedestal of 64 ADU was added to every pixel value. Initially the detector distance was fixed to 958.5 mm (determined using powder diffraction from an aluminium powder calibration grid). For **[1-NBD][S-BAr^F$_4$]**, 4 datasets from 4 crystals were combined to give 96.6% complete data to 0.95Å resolution. For **[1-NBA][S-BAr^F$_4$]**, 9 datasets from 9 crystals could be combined resulting in 94.0% complete data to 0.95Å resolution. The strong reflections from each of the combined datasets were used to post-refine the detector distance and unit cell parameters of each dataset. The mean refined detector distances for both datasets (**[1-NBD][S-BAr^F$_4$]**: 956.87(22) mm, **[1-NBA][S-BAr^F$_4$]:** 958.33(24) were within 0.2% of the initial estimate. The unit cell parameters for each of the combined datasets were then refined by fitting calculated to observed 2θ values.

The structures were solved *ab initio* using SHELXT.[8] Structure refinement was performed using SHELXL.[5] Electron scattering factors from Peng[9] were used in refinement. Anisotropic ADPs were refined for all non-hydrogen atoms and all hydrogen atoms were geometrically placed using the idealised (inter-nuclear) X-H distances used in refinement of structures against neutron diffraction data with SHELXL[10] and allowed to ride on their parent atoms. For **[1-NBA][S-BAr^F$_4$]**, the isotropic ADPs of hydrogens attached to C1, C2, C4 and C5 were set to 5x that of the attached carbon atom to reflect any uncertainty in the modelled position. S-F distances in the SF_5 groups were restrained to be equal, these were the only distance restraints applied. A combination of rigid-body restraints where the components of the ADPs in the direction of the bond are restrained to be equal (DELU instruction), thermal similarity restraints where the ADPs of spatially close atoms are restrained to have the same U(ij) components (SIMU instruction) and enhanced rigid-body restraints where the relative motion of a bonded pair of atoms is restrained to be perpendicular to the bond between them (RIGU instruction[11]) were applied to fragments of the structure. These restraints, together with refinement of an extinction parameter (EXTI instruction), enabled anisotropic refinement of all non-hydrogen atoms without resorting to use of ISOR or XNDP instructions to prevent ADPs of some atoms becoming non-positive definite during refinement.

S.3.3 [1-(NBD)][S-BAr$^F{}_4$]

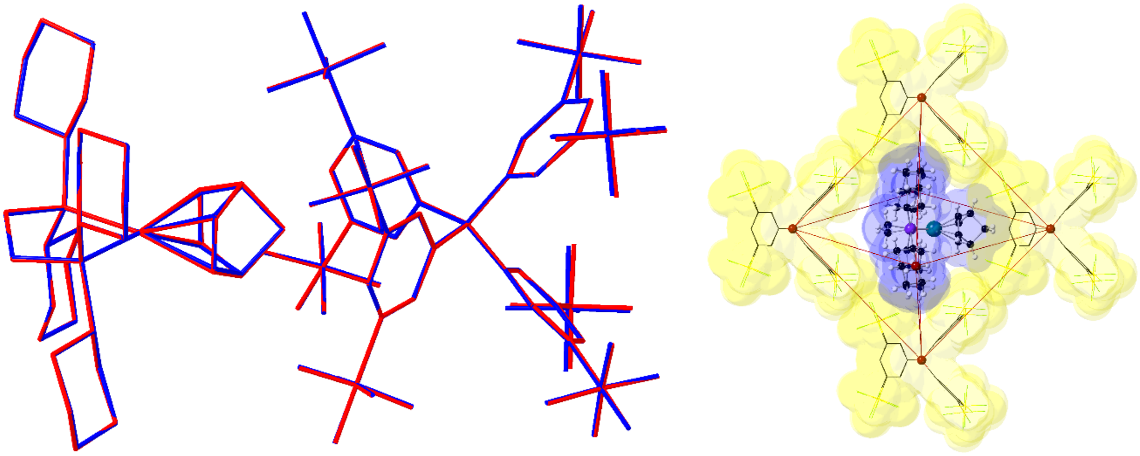

Figure S19. Left: overlay of the single-crystal X-ray (blue) and microED (red) structures of **[1-(NBD)][S-BAr$^F{}_4$]**. Right (X-ray): octahedral arrangement of anions around a single cation.

Table S3: Selected crystallographic and refinement data for [1-(NBD)][S-BAr$^F{}_4$].

Method	XRD	microED
Empirical formula	$C_{57}H_{68}BF_{40}P_2RhS_8$	$C_{57}H_{68}BF_{40}P_2S_8Rh$
Formula weight	1945.25	1945.25
Temperature/K	110(2)	80(2)
Crystal system	monoclinic	monoclinic
Space group	C2/c	C2/c
a/Å	19.5793(9)	19.603(3)
b/Å	18.3333(6)	18.393(3)
c/Å	20.4528(7)	20.438(2)
α/°	90	90
β/°	92.460(4)	92.355(12)
γ/°	90	90
Volume/Å^3	7334.8(5)	7362.8(18)
Z	4	4
ρ_{calc}g/cm^3	1.762	1.755
μ/mm^{-1}	5.723	-
F(000)	3912.0	1261.0

Crystal size	0.071 × 0.055 × 0.041 mm^3	microcrystals < ~1-2 μm
Radiation	Cu Kα (λ = 1.54184)	200 keV electron
Index ranges	-23 ≤ h ≤ 22, -13 ≤ k ≤ 22, -24 ≤ l ≤ 20	-20 ≤ h ≤ 20, -19 ≤ k ≤ 19, -21 ≤ l ≤ 21
Reflections collected	13332	19560
Independent reflections	6921 [R_{int} = 0.0554, R_{sigma} = 0.0880]	4380 [R_{int} = 0.2348, R_{sigma} = 0.1680]
Data/restraints/parameters	6921/402/548	4380/585/494
Goodness-of-fit on F^2	1.025	1.718
Final R indexes [I>=2σ (I)]	R_1 = 0.0660, wR_2 = 0.1627	R_1 = 0.1565, wR_2 = 0.3421
Final R indexes [all data]	R_1 = 0.1024, wR_2 = 0.1834	R_1 = 0.2194, wR_2 = 0.3584
Largest diff. peak/hole	1.36/-1.14 e $Å^{-3}$	0.25/-0.19 e $Å^{-1}$
CCDC no.	2126936	2126160

S.3.4 [1-(NBA)][S-BAr$^F{}_4$]

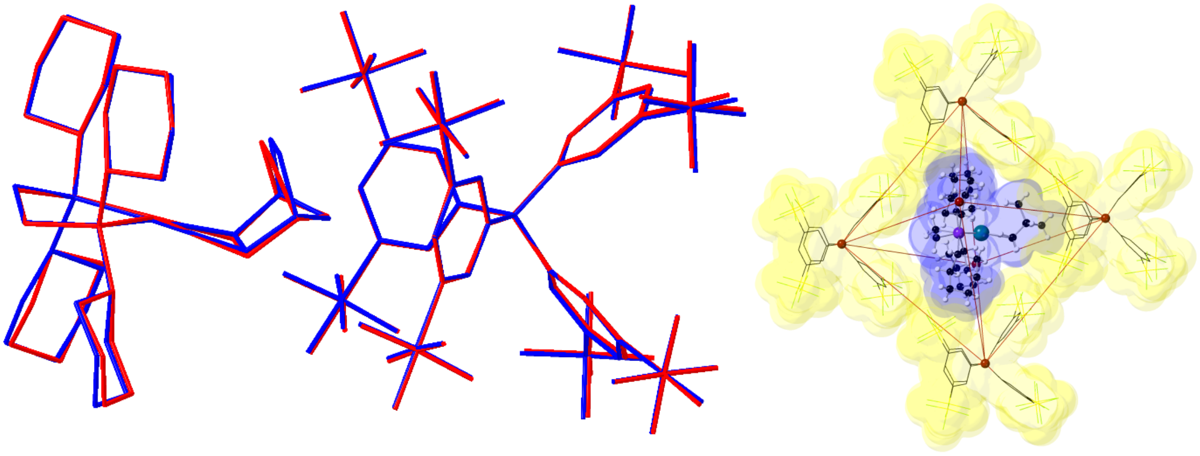

Figure S20. Left: overlay of the single-crystal X-ray (blue) and microED (red) structures of **[1-(NBA)][S-BAr$^F{}_4$]**. Right (X-ray): octahedral arrangement of anions around a single cation.

NBA binding pockets within **[1-(NBA)][S-BAr$^F{}_4$]** and **[1-(NBA)][BAr$^F{}_4$]** were estimated in the Olex2[6] program by calculating the solvent accessible void space (1.41 Å probe radius) within

the NBA-subtracted “[(dcpe)Rh]$^{+}$”[S-BAr$^{F}_{4}$]$^{-}$ and “[(dcpe)Rh]$^{+}$”[BAr$^{F}_{4}$]$^{-}$ structures, using Van der Waal radii taken from Alvarez.[12] Accordingly, for **[1-(NBA)][S-BAr$^{F}_{4}$]** and **[1-(NBA)][BAr$^{F}_{4}$]**, the pocket volumes were calculated to be 82 Å^{3} and 140 Å^{3}, respectively. Visual representations of the binding pockets were made using the CrystalMaker® program[13] (Figure S21).

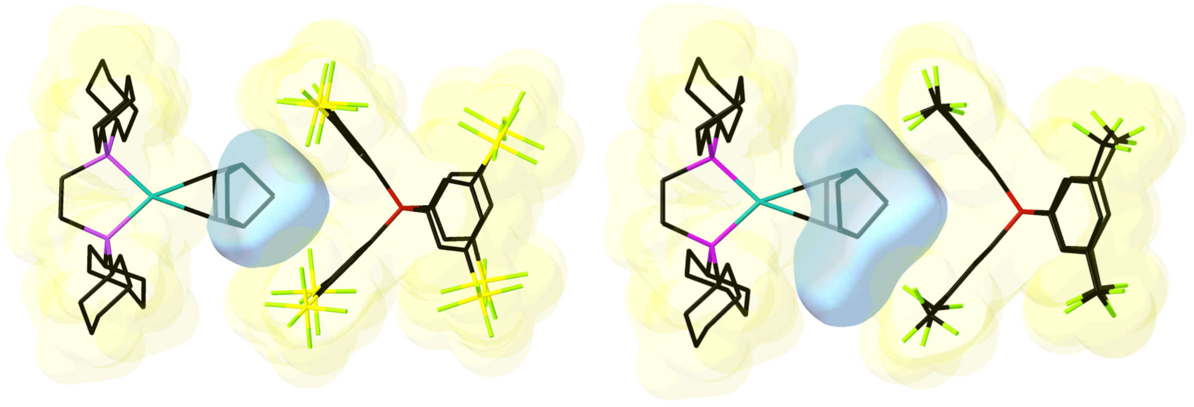

Figure S21. NBA binding pocket (blue surface) within the Van der Waal surfaces (yellow) of “[(dcpe)Rh]$^{+}$”[anion]$^{-}$ within **[1-(NBA)][S-BAr$^{F}_{4}$]** (left) and **[1-(NBA)][BAr$^{F}_{4}$]** (right).

Table S4: Selected crystallographic and refinement data for [1-(NBA)][S-BAr$^{F}_{4}$].

Method	XRD	microED
Empirical formula	$C_{57}H_{72}BF_{40}P_2S_8Rh$	$C_{57}H_{72}BF_{40}P_2S_8Rh$
Formula weight	1949.28	1949.29
Temperature/K	110(2)	80(2)
Crystal system	monoclinic	monoclinic
Space group	$P2_1/n$	$P2_1/n$
a/Å	19.4479(9)	19.5339(11)
b/Å	19.0194(10)	19.0160(14)
c/Å	20.1725(7)	20.3007(9)
α/°	90	90
β/°	91.572(4)	91.727(5)
γ/°	90	90
Volume/Å^{3}	7458.7(6)	7537.4(8)
Z	4	4

ρ_{calc}g/cm^3	1.736	1.718
μ/mm^{-1}	5.628	-
F(000)	3928.0	1269.0
Crystal size/mm^3	0.13 × 0.123 × 0.112	microcrystals < ~1-2 μm
Radiation	Cu Kα (λ = 1.54184)	200 keV electron
Index ranges	-22 ≤ h ≤ 23, -22 ≤ k ≤ 23, -24 ≤ l ≤ 15	-20 ≤ h ≤ 20, -19 ≤ k ≤ 19, -21 ≤ l ≤ 21
Reflections collected	26754	35027
Independent reflections	13926 [R_{int} = 0.0599, R_{sigma} = 0.0851]	8652 [R_{int} = 0.1906, R_{sigma} = 0.1786]
Data/restraints/parameters	13926/0/982	8652/1470/983
Goodness-of-fit on F^2	1.070	1.671
Final R indexes [I>=2σ (I)]	R_1 = 0.0865, wR_2 = 0.2273	R_1 = 0.1640, wR_2 = 0.3631
Final R indexes [all data]	R_1 = 0.1385, wR_2 = 0.2587	R_1 = 0.2369, wR_2 = 0.3800
Largest diff. peak/hole	1.68/-0.65 e Å^{-3}	0.25/-0.23 e Å^{-1}
CCDC no.	2126937	2126161

S.3.5 [1-(ethene)$_2$][S-BAr$^F_4$]

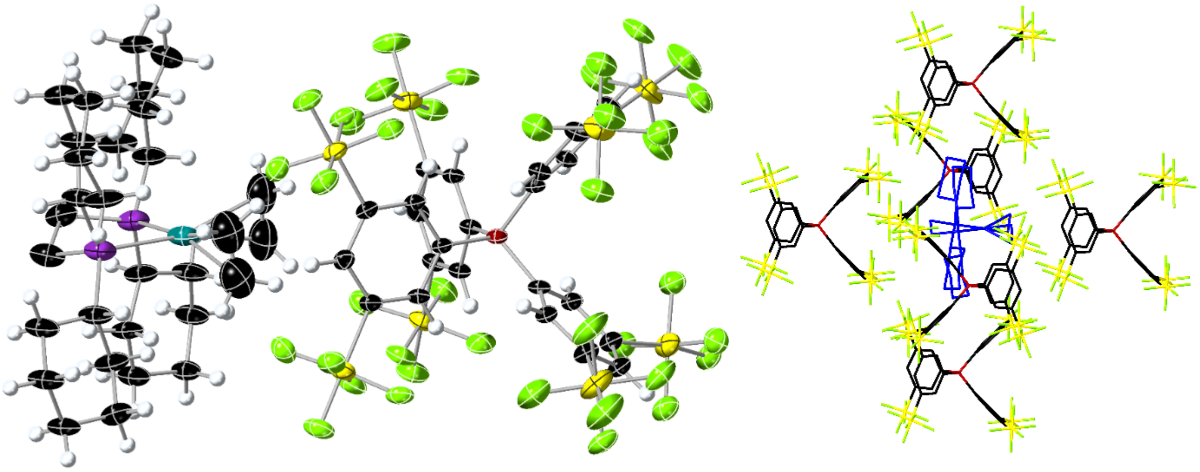

Figure S22. Structure and octahedral anion framework of **[1-(ethene)$_2$][S-BAr$^F_4$]**. Recorded at 110 K; ellipsoids at 25% probability. Selected bond distances (Å): Rh1-C1, 2.248(16); Rh1-C2, 2.256(16); R1-P1, 2.312(3); C1-C2, 1.25(3).

In the model, the cation is disordered over two positions, centred on Rh1 and Rh1A, which are related by a 180 degree rotation of the cation in the Rh1-P1-P2 plane; their respective occupancies were allowed to freely refine, converging at a final ratio of 0.844(4):0.156(4). The P(cyclohexyl)$_2$ groups are shared between the two orientations, however the ethene and diphosphine ligand $(CH_2)_2$ backbone could only be located for the major orientation. The residual electron density from the minor orientation has therefore been accounted for by incorporation into the major orientation.

Table S5: Selected crystallographic and refinement data for [1-(ethene)$_2$][S-BAr$^F_4$].

Method	XRD
Empirical formula	$C_{54}H_{64}BF_{40}P_2RhS_8$
Formula weight	1905.19
Temperature/K	110(2)
Crystal system	monoclinic
Space group	C2/c
a/Å	19.5254(8)
b/Å	18.4417(19)

c/Å	20.4850(10)
α/°	90
β/°	92.333(4)
γ/°	90
Volume/Å^3	7370.2(9)
Z	4
ρ_{calc}g/cm^3	1.717
μ/mm^{-1}	5.681
F(000)	3824.0
Crystal size/mm^3	0.22 × 0.214 × 0.117
Radiation	Cu Kα (λ = 1.54184)
2Θ range for data collection/°	7.784 to 141.576
Index ranges	-14 ≤ h ≤ 23, -22 ≤ k ≤ 21, -24 ≤ l ≤ 24
Reflections collected	13346
Independent reflections	6905 [R_{int} = 0.0351, R_{sigma} = 0.0431]
Data/restraints/parameters	6905/858/648
Goodness-of-fit on F^2	1.090
Final R indexes [I>=2σ (I)]	R_1 = 0.0946, wR_2 = 0.2460
Final R indexes [all data]	R_1 = 0.1319, wR_2 = 0.2755
Largest diff. peak/hole / e Å^{-3}	1.58/-0.64
CCDC no.	2126935

S.4 COMPUTATIONAL METHODS

Periodic DFT calculations on **[1-NBA][S-BAr$^F_4$]** employed the CP2K program suite (Version 5.0).[14] Initial coordinates were obtained from the experimental crystallographic data, with the

hydrogen positions normalised with Mercury.[15] Calculations employed the Gaussian Plane Wave (GPW) formalism as implemented in the QUICKSTEP[16] module with basis sets for all atoms of double-ζ plus polarization quality in their short-range variant (DZVP-MOLOPT-SR-GTH).[17] The interaction between the core electrons and the valence shell (Rh: 17, B: 3, C: 4, P: 5, F: 7, H: 1 electrons) was described by Goedecker-Teter-Hutter (GTH) pseudo potentials.[18] The generalized gradient approximation (GGA) to the exchange-correlation functional according to Perdew-Burke-Ernzerhof (PBE)[19] was used in combination with Grimme's D3-correction for dispersion interactions.[20] The auxiliary plane wave basis set was truncated at a cutoff of 500 Ry. The maximum force convergence criterion was set to 10^{-4} Eh·Bohr^{-1}, whilst default values were used for the remaining criteria. The convergence criterion for the self-consistent field (SCF) accuracy was set to 10^{-7} Eh and 10^{-8} Eh for geometry optimizations and vibrational analysis, respectively.

The Brillouin zone was sampled using the Γ-point. Periodic boundary conditions (PBC) were applied throughout in combination with fixed unit cell parameters obtained from experiment. All geometries were first partially relaxed, keeping the heavy atoms (non-H, F) fixed, then fully relaxed without imposing any constraints, whilst keeping unit cell parameters constant in all cases. The fully optimised geometry was further characterized by analysis of the numerical second derivatives with a displacement of 0.01 Bohr and found to have no imaginary eigenvalues. Cartesian coordinates of computed structure are included below.

All interaction energy calculations were performed in a periodic box of the same dimension as the **[1-NBA][S-BAr$^F_4$]** unit cell. Test calculations showed that increasing the size of the periodic box did not significantly affect the energies. For the lattice energies, geometries for the **[1-NBA]$^+$** cation and **[S-BAr$^F_4$]$^-$** anion were taken from the fully optimised unit cell. The normalised lattice energy quoted in the main text is the total lattice energy divided by Z, the number of formula units in the unit cell (here 4). Incorporation energies, ΔE_1, were calculated by removing one NBA ligand from the full unit cell and recomputing the electronic energy. Molecular interaction energies, ΔE_2, correspond to the change in electronic energy upon removing the NBA ligand from **[1-NBA]$^+$**.

Geometries for the electronic structure analyses were extracted from the optimised CP2K geometry. The topology of the electron density of the **[1-NBA]$^+$** cation was analysed by means of QTAIM (Quantum Theory of Atoms in Molecules),[21] as implemented in the AIMALL package.[22] Inner shell electrons on Rh and P modelled by ECPs were represented by core density functions (extended wavefunction format). NBO calculations were performed using the NBO 6.0 program.[23] NCI calculations on the nearest neighbour [1-NBA][S-BAr$^F_4$] ion-pair were performed using the NCIPLOT program.[24] The promolecular electron density was employed. Orbital plots were created with Chemcraft[25] with an outer contour value of 0.07465.

Short inter-ion contacts were analysed using the Crystal Explorer package,[26] using a central cation and the six nearest neighbour anions.

S.4.1 QTAIM study of [1-NBA]$^+$

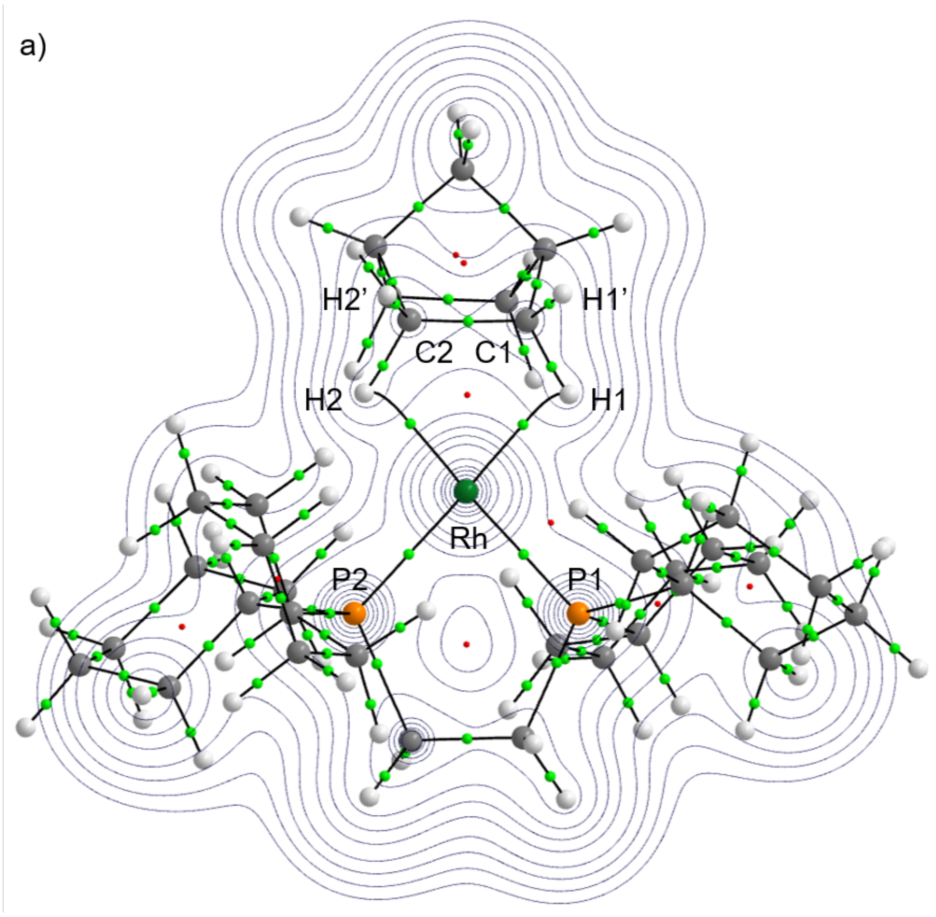

(b)

Bond	Distance/Å	ρ(r)	∇^2□(r)	ε	H(r)
**Rh–H1**	1.882	0.060	0.246	1.208	-0.008
**Rh–H2**	1.882	0.060	0.246	1.229	-0.008
**C1–H1**	1.153	0.233	-0.615	0.013	-0.203
**C1–H1'**	1.097	0.277	-0.946	0.003	-0.279
**C2–H2**	1.153	0.234	-0.619	0.014	-0.204
**C2–H2'**	1.095	0.278	-0.957	0.002	-0.282
**Rh–P1**	2.209	0.119	0.077	0.014	-0.059
**Rh–P2**	2.207	0.119	0.078	0.005	-0.099
**C1–C2**	1.562	0.228	-0.457	0.011	-0.172

Figure S23. (a) Molecular graph for the **[1-NBA]**$^+$ cation with bond critical bonds (BCPs) in green and ring critical points (RCPs) in red. Electron density contours are shown in the plane containing Rh, H1 and H2 (b) Selected metrics for key BCPs (atomic units unless otherwise stated).

S.4.2 Non-covalent interaction (NCI) study of the [1-NBA][S-BAr$^F{}_4$] ion-pair

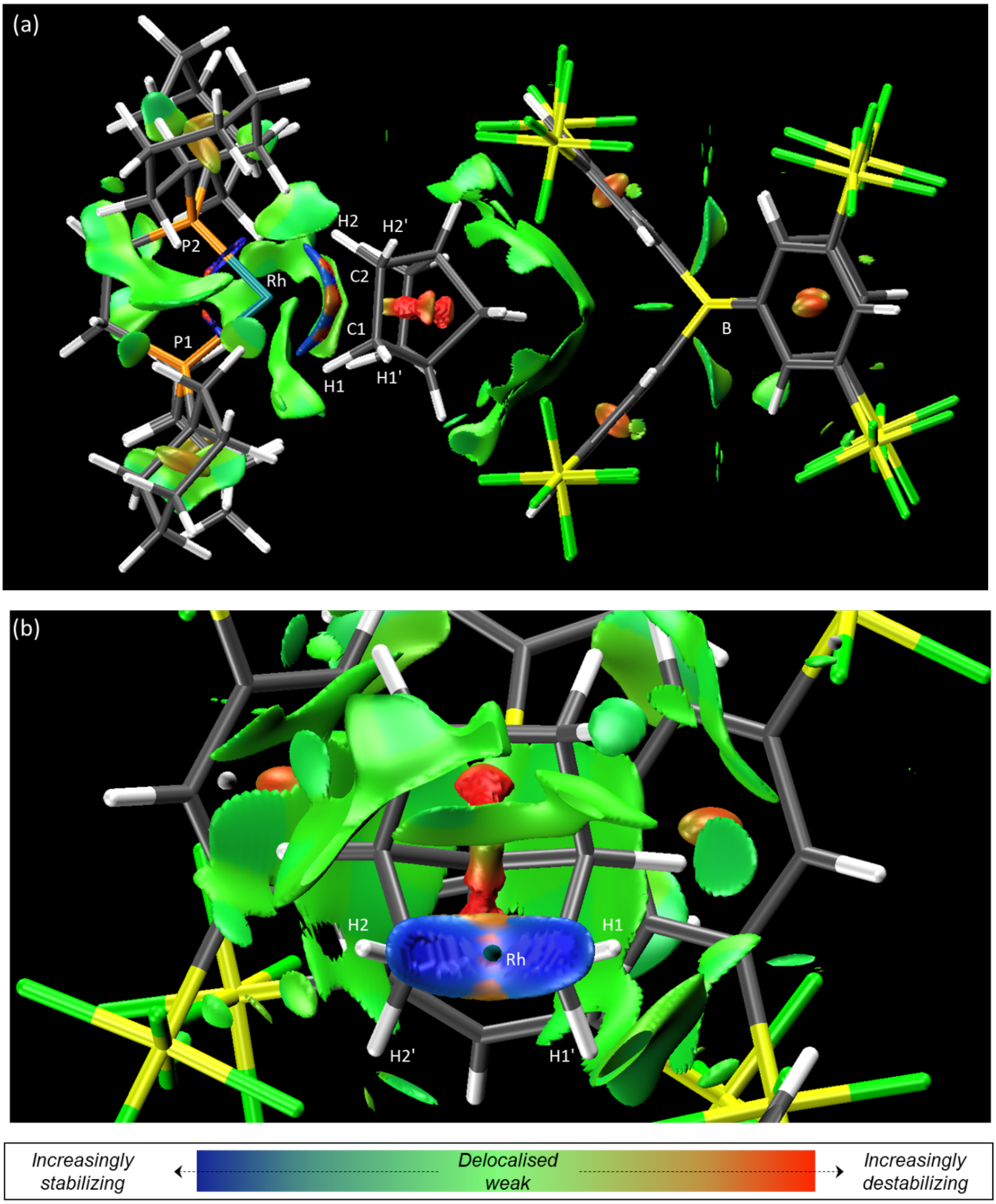

Figure S24. Two views of the NCI plots for the [1-NBA][S-BAr$^F{}_4$] ion-pair (a) View from above the NBA ligand showing both the cation and anion (b) Detail viewed from the Rh center looking down an axis passing through the center of the C1-C1' bond. Isosurfaces are generated for $\sigma = 0.3$ au and $-0.07 < \rho < 0.07$ au; a key showing the color scheme employed is also provided.

S.4.3 Natural bond orbital analysis of the [1-NBA]$^+$ cation

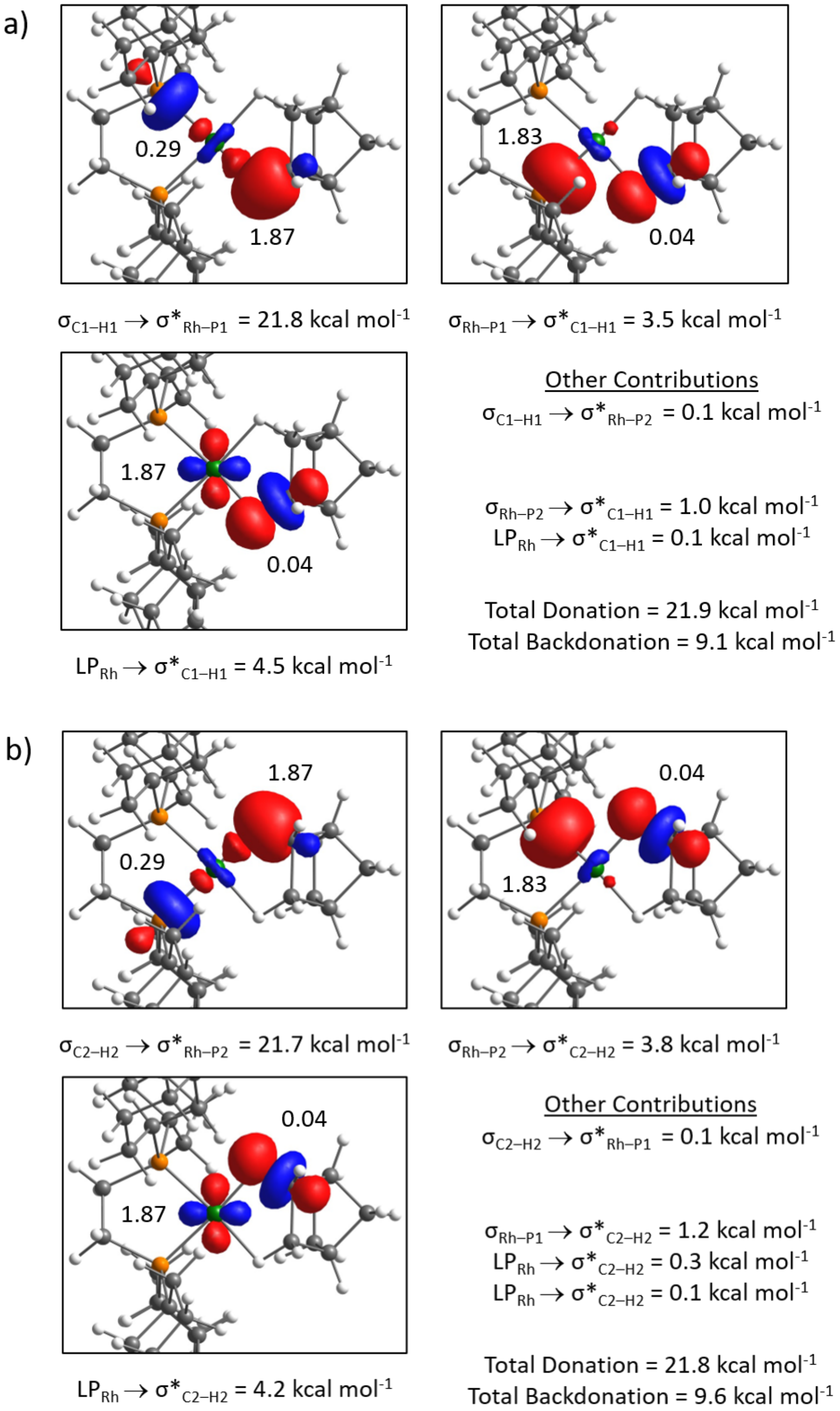

Figure S25. Major donor−acceptor interactions in **[1-NBA]$^+$** derived from the second-order perturbation NBO analysis (kcal/mol); (a) involving C1-H1; (b) involving C2-H2. NBO occupations are also indicated and well as other minor contributions to the back donations.

S.4.4 CrystalExplorer analysis of [1-NBA][S-BAr$^F{}_4$] and [1-NBA][BAr$^F{}_4$]

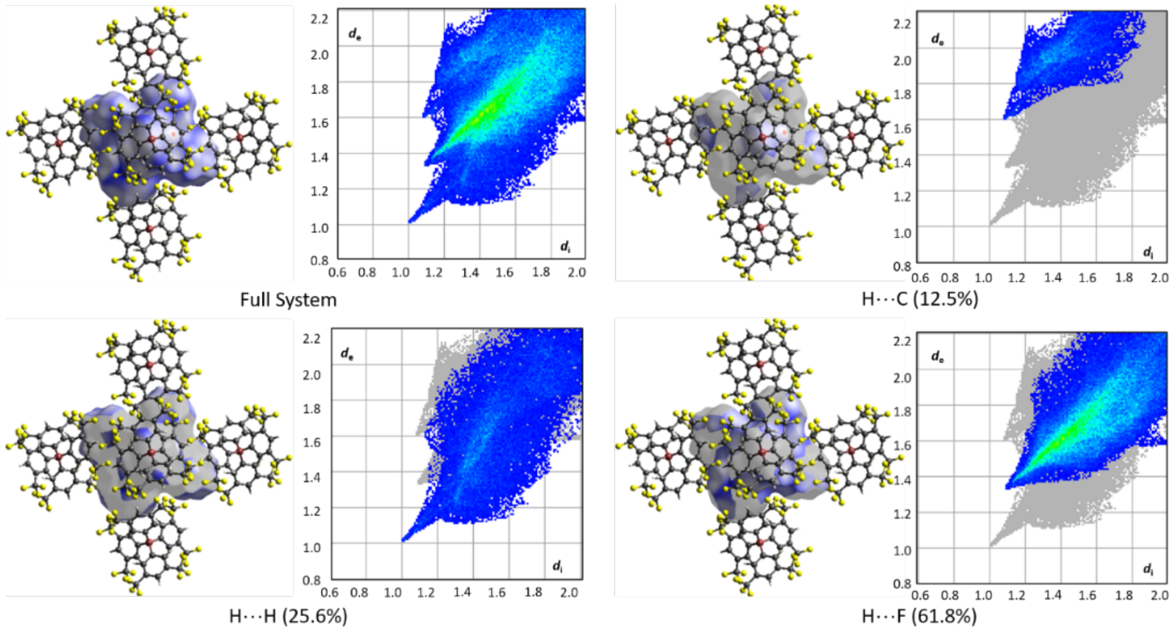

Figure S26. Hirshfeld surfaces plotted around **[1-NBA]$^+$** in **[1-NBA][BAr$^F{}_4$]** with accompanying fingerprint plots broken down into the three types of anion-cation contacts.

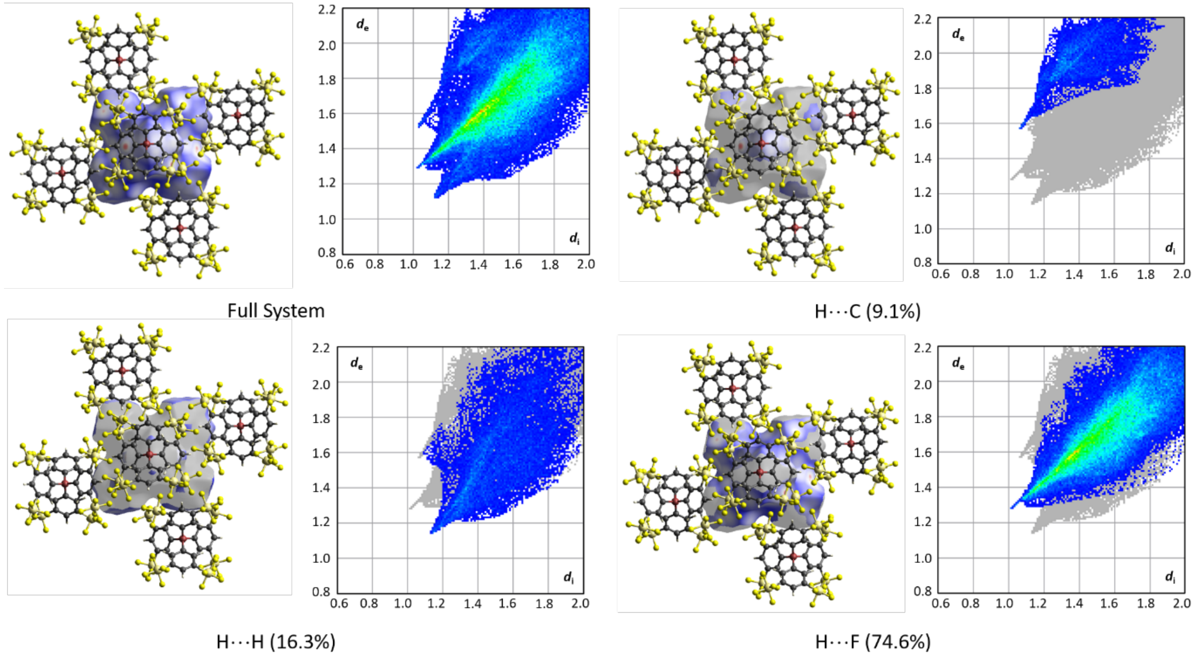

Figure S27. Hirshfeld surfaces plotted around **[1-NBA]$^+$** in **[1-NBA][S-BAr$^F{}_4$]** with accompanying fingerprint plots broken down into the three types of anion-cation contacts.

[1-NBA][S-BAr$^F_4$] **[1-NBA][BAr$^F_4$]**

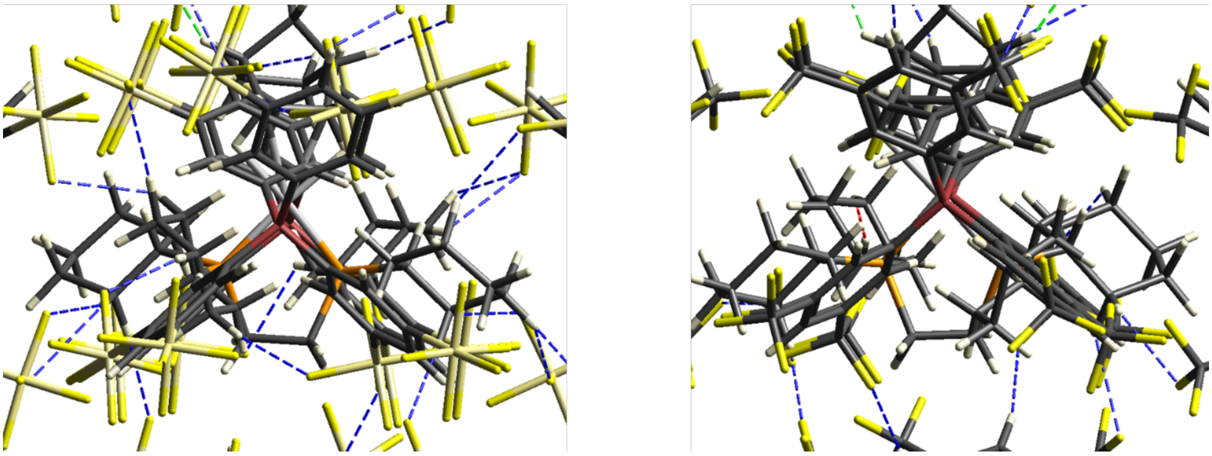

Figure S28. Close up of the **[1-NBA]$^+$** environments in **[1-NBA][S-BAr$^F_4$]** and **[1-NBA][BAr$^F_4$]** highlighting short contacts at or below the sum of the van der Waals radii (blue: C–H···F–C; red: C–H···H–C; green: C–H···C).

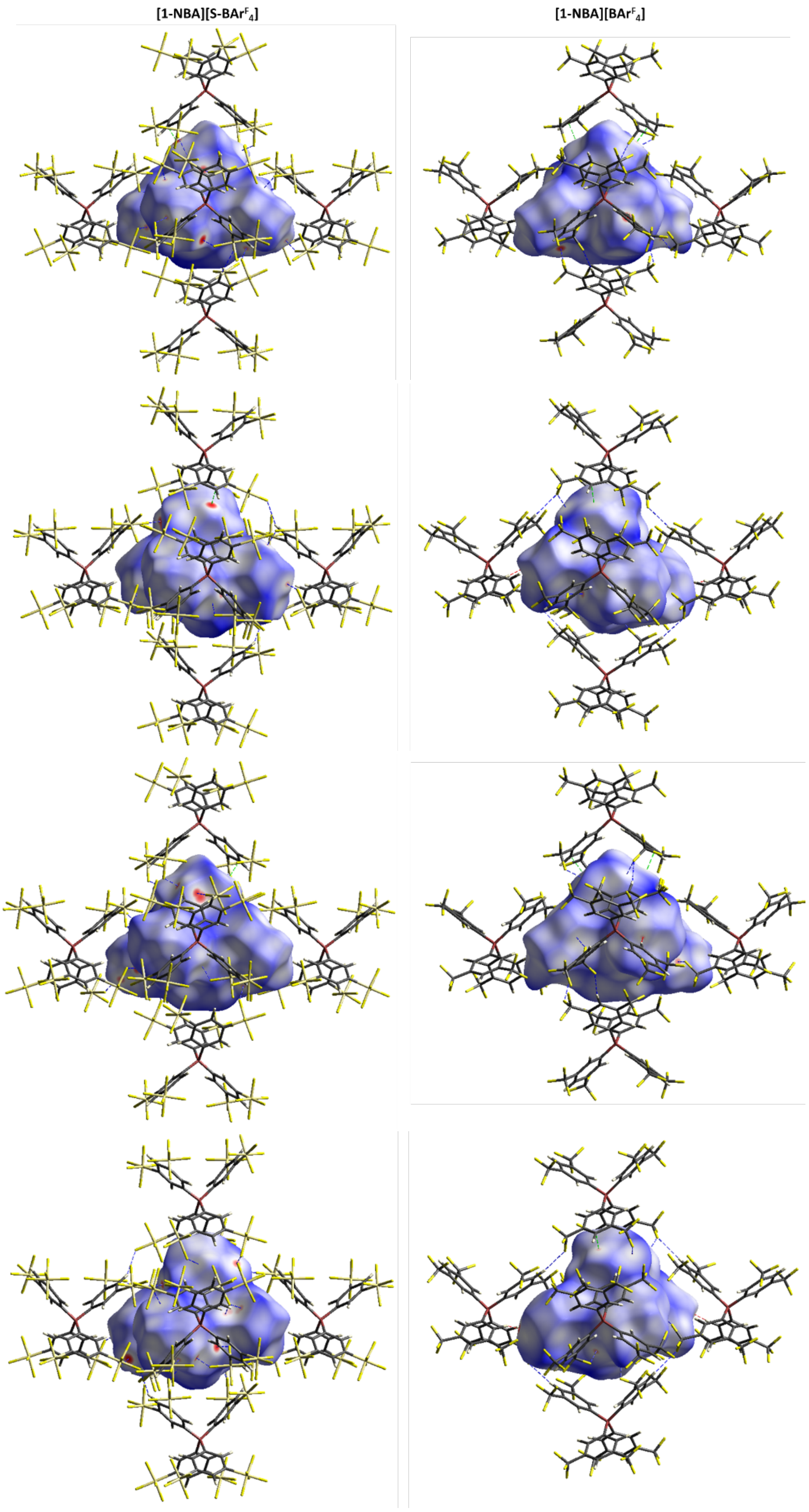

Figure S29. Hirshfeld surfaces of the **[1-NBA]$^+$** cation within the pseudo-octahedral anion environments in **[1-NBA][S-BAr^F$_4$]** and **[1-NBA][BAr^F$_4$]**; a view from behind each equatorial anion is presented. Red areas are contacts below, white at, and blue greater than the sum of the van der Waals radii.

S.4.5 Computed cartesian coordinates (Å) and energy for [1-NBA][S-BAr$^F{}_4$]

SCF Energy = -6178.989205

Rh	18.832286	13.501740	15.023018
P	19.385817	15.175488	13.692151
P	18.648607	15.044944	16.590486
C	19.040509	16.826705	14.481895
H	19.689586	17.600330	14.048299
H	18.003548	17.080246	14.219920
C	19.205768	16.723177	16.008228
H	18.658339	17.514164	16.540017
H	20.262506	16.816488	16.294244
C	18.368535	15.041992	12.149548
H	18.696954	14.065724	11.750326
C	16.862147	14.920144	12.457613
H	16.697299	14.179132	13.255904
H	16.483699	15.881877	12.836214
C	16.095343	14.530835	11.185145
H	16.408972	13.523102	10.875731
H	15.020986	14.463671	11.401532
C	16.350749	15.523896	10.044101
H	15.904990	16.497197	10.297738
H	15.846713	15.183275	9.128461
C	17.852390	15.709732	9.782232
H	18.007150	16.479337	9.015196
H	18.275780	14.772251	9.386210
C	18.617676	16.098927	11.062367
H	19.686920	16.194570	10.847675
H	18.275620	17.081455	11.414436
C	21.168987	15.248010	13.140349
H	21.293337	16.228442	12.650189
C	21.498216	14.139538	12.121980
H	20.875464	14.245864	11.226556
H	21.253633	13.158700	12.558874
C	22.983665	14.155096	11.718458
H	23.179821	13.336016	11.013948
H	23.204725	15.088926	11.180577
C	23.898623	14.038868	12.940882
H	23.752849	13.058385	13.419657
H	24.952894	14.072998	12.627662
C	23.597387	15.153688	13.946803
H	23.869163	16.127256	13.513267
H	24.208895	15.031743	14.850912
C	22.115019	15.166644	14.356935
H	21.934141	16.002998	15.046222
H	21.877871	14.246975	14.913468
C	19.618426	14.645546	18.123846
H	19.130893	13.722752	18.483937
C	19.533069	15.699841	19.241201
H	18.490688	15.937034	19.475577
H	20.003736	16.634520	18.904573
C	20.244784	15.198862	20.511576
H	20.206763	15.985686	21.279308
H	19.693987	14.334282	20.913267
C	21.699738	14.794460	20.234821
H	22.156322	14.378775	21.144354
H	22.283887	15.690681	19.979889
C	21.788781	13.781713	19.083998
H	22.838943	13.544528	18.861554
H	21.317114	12.834950	19.387010
C	21.089000	14.304387	17.819741
H	21.609843	15.203649	17.459164
H	21.144214	13.566003	17.006546
C	16.900723	15.232614	17.201196
H	16.968277	15.800215	18.143347
C	15.980271	16.010739	16.242545
H	16.373050	17.019954	16.052818
H	15.944467	15.490898	15.272914
C	14.560562	16.115208	16.831535
H	14.595148	16.711771	17.755346
H	13.915882	16.666568	16.137258
C	13.970330	14.733127	17.140766
H	13.844503	14.166745	16.205167
H	12.971253	14.834311	17.586496
C	14.887621	13.953166	18.089574

H	14.490489	12.948230	18.285438
H	14.931118	14.463761	19.063221
C	16.300294	13.842922	17.499987
H	16.949003	13.271081	18.174623
H	16.250080	13.282596	16.553450
Rh	9.482615	4.012721	15.166050
P	8.929196	5.686390	16.497048
P	9.666302	5.556070	13.598738
C	9.274632	7.337670	15.707476
H	8.625623	8.111309	16.141147
H	10.311619	7.591089	15.969474
C	9.109392	7.234328	14.181137
H	9.656963	8.025279	13.649443
H	8.052678	7.327831	13.895100
C	9.946522	5.552825	18.039605
H	9.618218	4.576503	18.438785
C	11.452905	5.431142	17.731423
H	11.617762	4.690105	16.933156
H	11.831228	6.392879	17.352695
C	12.219864	5.041985	19.003845
H	11.906418	4.034215	19.313320
H	13.294222	4.974993	18.787406
C	11.964377	6.035017	20.144899
H	12.409894	7.008429	19.891228
H	12.468577	5.694518	21.060493
C	10.462727	6.220586	20.406908
H	10.307883	6.990142	21.173976
H	10.039542	5.283014	20.802932
C	9.697304	6.609688	19.126837
H	8.628060	6.705170	19.341606
H	10.039197	7.592277	18.774777
C	7.146037	5.758956	17.048908
H	7.021729	6.739321	17.539212
C	6.816788	4.650334	18.067104
H	7.439536	4.756517	18.962547
H	7.061374	3.669566	17.630052
C	5.331336	4.665822	18.470627
H	5.135176	3.846619	19.174991
H	5.110282	5.599557	19.008672
C	4.416371	4.549815	17.248187
H	4.562144	3.569420	16.769229
H	3.362101	4.583878	17.561427
C	4.717612	5.664820	16.242474
H	4.445876	6.638314	16.676200
H	4.106085	5.543075	15.338351
C	6.199976	5.677775	15.832339
H	6.380888	6.514183	15.143130
H	6.437071	4.758149	15.275721
C	8.696137	5.156873	12.065598
H	9.183082	4.233711	11.705647
C	8.781891	6.210829	10.947955
H	9.824368	6.447299	10.713285
H	8.311895	7.145894	11.284447
C	8.069608	5.709978	9.677851
H	8.107889	6.496622	8.909947
H	8.619827	4.845000	9.276216
C	6.614506	5.306439	9.955058
H	6.157460	4.890831	9.045720
H	6.030900	6.203033	10.209910
C	6.525240	4.294006	11.106125
H	5.475033	4.057228	11.328756
H	6.996540	3.347053	10.803283
C	7.225456	4.816608	12.370108
H	6.705183	5.716212	12.730638
H	7.170053	4.078375	13.183432
C	11.414046	5.743473	12.987695
H	11.346330	6.310811	12.045401
C	12.334776	6.521838	13.945844
H	11.942104	7.531106	14.135488
H	12.371006	6.002289	14.915614
C	13.754198	6.626124	13.356188
H	13.719142	7.222170	12.432057
H	14.399101	7.177951	14.049814
C	14.344385	5.243919	13.047436
H	14.470433	4.677952	13.983262
H	15.343348	5.344905	12.601408
C	13.426842	4.463574	12.099190

H	13.823866	3.458537	11.903644
H	13.383146	4.973772	11.125347
C	12.014302	4.353649	12.689148
H	11.365381	3.781659	12.014833
H	12.064689	3.793600	13.635838
Rh	19.509627	5.476560	5.102966
P	18.956096	3.802813	6.433834
P	19.693305	3.933356	3.535500
C	19.301403	2.151595	5.644089
H	18.652327	1.377969	6.077686
H	20.338364	1.898055	5.906064
C	19.136144	2.255122	4.117757
H	19.683571	1.464136	3.585967
H	18.079405	2.161812	3.831741
C	19.973376	3.936307	7.976438
H	19.644957	4.912574	8.375660
C	21.479765	4.058156	7.668373
H	21.644612	4.799169	6.870083
H	21.858213	3.096424	7.289771
C	22.246569	4.447465	8.940841
H	21.932939	5.455197	9.250256
H	23.320925	4.514630	8.724454
C	21.991164	3.454402	10.081885
H	22.436923	2.481102	9.828246
H	22.495200	3.795023	10.997525
C	20.489523	3.268566	10.343754
H	20.334763	2.498961	11.110789
H	20.066133	4.206047	10.739775
C	19.724236	2.879371	9.063618
H	18.654992	2.783727	9.278311
H	20.066294	1.896844	8.711549
C	17.172926	3.730289	6.985636
H	17.048574	2.749856	7.475793
C	16.843696	4.838757	8.004007
H	17.466449	4.732429	8.899432
H	17.088281	5.819597	7.567117
C	15.358247	4.823201	8.407531
H	15.162093	5.642280	9.112043
H	15.137186	3.889370	8.945409
C	14.443290	4.939433	7.185107
H	14.589064	5.919918	6.706335
H	13.389019	4.905302	7.498327
C	14.744524	3.824616	6.179182
H	14.472747	2.851048	6.612716
H	14.133016	3.946564	5.275074
C	16.226892	3.811659	5.769050
H	16.407769	2.975306	5.079762
H	16.464042	4.731329	5.212519
C	18.723485	4.332755	2.002140
H	19.211018	5.255549	1.642050
C	18.808844	3.278460	0.884784
H	19.851225	3.041268	0.650410
H	18.338178	2.343781	1.221413
C	18.097128	3.779438	-0.385590
H	18.135150	2.992613	-1.153322
H	18.647925	4.644018	-0.787282
C	16.642174	4.183839	-0.108836
H	16.185590	4.599523	-1.018369
H	16.058026	3.287618	0.146097
C	16.553130	5.196585	1.041987
H	15.502968	5.433771	1.264430
H	17.024797	6.143349	0.738974
C	17.252912	4.673913	2.306244
H	16.732068	3.774652	2.666821
H	17.197697	5.412298	3.119439
C	21.441187	3.745685	2.924789
H	21.373635	3.178084	1.982638
C	22.361640	2.967561	3.883440
H	21.968860	1.958346	4.073168
H	22.397443	3.487402	4.853071
C	23.781348	2.863091	3.294451
H	23.746763	2.266527	2.370640
H	24.426028	2.311731	3.988729
C	24.371581	4.245171	2.985220
H	24.497408	4.811554	3.920819
H	25.370659	4.143988	2.539490
C	23.454291	5.025133	2.036412

H	23.851424	6.030069	1.840547
H	23.410794	4.514537	1.062765
C	22.041617	5.135376	2.625999
H	21.392909	5.707219	1.951363
H	22.091832	5.695702	3.572536
Rh	9.393898	14.965579	4.959937
P	9.947317	13.291910	3.628938
P	9.210211	13.422230	6.527248
C	9.601880	11.640631	4.418510
H	10.250889	10.866991	3.984839
H	8.564893	11.387211	4.156512
C	9.767120	11.743972	5.944849
H	9.219550	10.953021	6.476544
H	10.823835	11.650469	6.230886
C	8.929991	13.425475	2.086381
H	9.258296	14.401796	1.687201
C	7.423608	13.547159	2.394563
H	7.258752	14.288197	3.192830
H	7.045284	12.585423	2.773291
C	6.656650	13.936316	1.122140
H	6.970097	14.944086	0.812665
H	5.582292	14.003309	1.338580
C	6.912136	12.943283	-0.018912
H	6.466618	11.969872	0.234760
H	6.407935	13.283782	-0.934507
C	8.413786	12.757713	-0.280922
H	8.568629	11.988156	-1.047990
H	8.836972	13.695285	-0.676946
C	9.179208	12.368611	0.999150
H	10.248452	12.273129	0.784380
H	8.837315	11.386022	1.351210
C	11.730475	13.219343	3.077079
H	11.854783	12.238978	2.586776
C	12.059725	14.327963	2.058882
H	11.436977	14.221779	1.163438
H	11.815138	15.308733	2.495932
C	13.545177	14.312477	1.655359
H	13.741337	15.131679	0.950994
H	13.766232	13.378741	1.117314
C	14.460142	14.428486	2.877798
H	14.314370	15.408881	3.356755
H	15.514412	14.394421	2.564558
C	14.158901	13.313482	3.883513
H	14.430638	12.339988	3.449789
H	14.770428	13.435229	4.787636
C	12.676537	13.300526	4.293648
H	12.495625	12.464119	4.982857
H	12.439441	14.220152	4.850265
C	10.180376	13.821427	8.060388
H	9.693431	14.744589	8.420339
C	10.094621	12.767471	9.178032
H	9.052144	12.531002	9.412701
H	10.564617	11.832406	8.841539
C	10.806904	13.268322	10.448136
H	10.768623	12.481678	11.216039
H	10.256684	14.133300	10.849770
C	12.262006	13.671860	10.170930
H	12.719051	14.087468	11.080267
H	12.845612	12.775265	9.916077
C	12.351272	14.684293	9.019862
H	13.401480	14.921071	8.797232
H	11.879972	15.631246	9.322705
C	11.651057	14.161692	7.755879
H	12.171330	13.262087	7.395349
H	11.706460	14.899924	6.942555
C	7.462467	13.234827	7.138291
H	7.530182	12.667488	8.080585
C	6.541737	12.456463	6.180142
H	6.934409	11.447195	5.990497
H	6.505507	12.976013	5.210373
C	5.122314	12.352177	6.769797
H	5.157371	11.756130	7.693928
H	4.477412	11.800351	6.076170
C	4.532128	13.734382	7.078551
H	4.406079	14.300349	6.142726
H	3.533165	13.633396	7.524579
C	5.449672	14.514726	8.026797

H	5.052648	15.519763	8.222345
H	5.493367	14.004527	9.000640
C	6.862212	14.624650	7.436839
H	7.511132	15.196640	8.111154
H	6.811824	15.184699	6.490149
C	18.524546	11.327404	14.034166
H	17.634453	11.144680	13.419415
H	18.958557	12.296482	13.583689
C	18.168077	11.248467	15.552938
H	18.364919	12.148831	16.245450
H	17.100330	11.050550	15.691776
C	19.057044	10.094072	16.068738
H	18.690942	9.677328	17.009117
C	20.523414	10.560857	16.109756
H	21.151750	9.810812	16.603543
H	20.641253	11.501283	16.663496
C	20.887060	10.697256	14.598276
H	21.160404	11.726163	14.325841
H	21.727186	10.051514	14.330357
C	19.603830	10.234604	13.876261
H	19.757094	9.937520	12.835757
C	19.059663	9.155791	14.840876
H	18.063390	8.786817	14.567484
H	19.735771	8.300696	14.958936
C	9.790455	1.838172	16.154482
H	10.680600	1.655494	16.769169
H	9.356274	2.807101	16.605112
C	10.146853	1.759521	14.635675
H	9.949844	2.659981	13.943355
H	11.214605	1.561715	14.496732
C	9.257995	0.605112	14.119709
H	9.624119	0.188554	13.179251
C	7.791577	1.071782	14.078904
H	7.163262	0.321838	13.584939
H	7.673636	2.012367	13.525444
C	7.427999	1.207732	15.590446
H	7.154447	2.236506	15.863171
H	6.588031	0.561734	15.858246
C	8.711364	0.745161	16.312273
H	8.558227	0.447859	17.352733
C	9.255609	-0.333385	15.347402
H	10.251977	-0.702265	15.620615
H	8.579636	-1.188571	15.229259
C	19.817369	7.650895	6.091817
H	20.707462	7.833617	6.706568
H	19.383357	6.681816	6.542293
C	20.173835	7.729833	4.573045
H	19.976991	6.829469	3.880533
H	21.241581	7.927750	4.434205
C	19.284868	8.884230	4.057247
H	19.650970	9.300975	3.116868
C	17.818499	8.417445	4.016230
H	17.190162	9.167491	3.522444
H	17.700659	7.477020	3.462488
C	17.454854	8.281044	5.527710
H	17.181511	7.252137	5.800144
H	16.614728	8.926786	5.795631
C	18.738085	8.743694	6.249725
H	18.584822	9.040776	7.290229
C	19.282251	9.822509	5.285110
H	20.278525	10.191482	5.558500
H	18.606144	10.677604	5.167052
C	9.086056	17.140128	3.971505
H	8.195911	17.322804	3.356818
H	9.520238	16.171199	3.520875
C	8.729660	17.218779	5.490312
H	8.926668	16.318319	6.182631
H	7.661907	17.416586	5.629256
C	9.618517	18.373188	6.006278
H	9.252394	18.789747	6.946736
C	11.084935	17.906518	6.047082
H	11.713250	18.656462	6.541047
H	11.202876	16.965933	6.600542
C	11.448513	17.770568	4.535540
H	11.722065	16.741794	4.262816
H	12.288481	18.416567	4.267739
C	10.165148	18.233138	3.813713

H	10.318284	18.530439	2.773253
C	9.620903	19.311685	4.778584
H	8.624535	19.680564	4.505372
H	10.296875	20.166871	4.896726
S	5.504722	10.381145	16.633443
S	7.051959	10.777216	11.340792
S	7.630980	17.044153	10.855191
S	12.819032	17.315917	12.779483
S	13.752735	11.319369	13.658147
S	11.585236	10.809394	18.710975
S	5.487737	16.881595	17.737267
S	10.788421	17.136918	19.236290
F	6.826684	10.378652	17.612807
F	5.946117	8.895862	16.074362
F	4.153317	10.303974	15.720448
F	4.978035	11.793067	17.263239
F	4.674467	9.634744	17.818522
F	7.083930	9.199282	11.803626
F	8.683360	10.746699	11.179923
F	7.035544	12.313300	10.762455
F	5.425322	10.752252	11.390451
F	6.929305	10.296024	9.786301
F	7.182980	15.490707	10.616190
F	8.663599	16.970927	9.572680
F	8.036973	18.619020	11.040429
F	6.499423	17.198211	12.022947
F	6.491029	17.495171	9.787609
F	13.529493	15.865893	13.070857
F	12.777141	17.642105	14.391880
F	12.250965	18.828950	12.507158
F	12.939479	17.052542	11.175772
F	14.326646	17.939365	12.724030
F	14.925729	11.421827	14.811090
F	13.962811	12.900449	13.279786
F	12.680513	11.188430	12.437176
F	13.644971	9.713897	13.972085
F	14.920252	10.970999	12.579071
F	11.658404	9.277008	18.102214
F	13.216062	10.894514	18.799571
F	11.473439	12.297439	19.424538
F	9.961341	10.674607	18.731336
F	11.594233	10.156654	20.197689
F	4.957704	15.343442	17.542117
F	5.360409	17.126277	16.127389
F	5.895604	18.457944	17.897302
F	5.493013	16.681427	19.358154
F	3.938234	17.345077	17.885115
F	11.477170	15.651863	19.358720
F	9.921746	16.832751	20.610210
F	10.180746	18.672298	19.154515
F	11.734749	17.493249	17.956070
F	11.956621	17.717597	20.196880
C	8.078739	12.933316	14.685876
C	7.271127	12.312779	15.657768
H	7.307732	12.656457	16.688711
C	6.452501	11.235169	15.319210
C	6.360490	10.740926	14.021125
H	5.708345	9.912955	13.767830
C	7.157475	11.368609	13.066228
C	8.008477	12.426562	13.374471
H	8.632984	12.858073	12.596448
C	9.573430	15.079356	13.897653
C	8.597113	15.549210	13.001538
H	7.582821	15.162151	13.059860
C	8.922195	16.499189	12.032858
C	10.201690	17.034091	11.904620
H	10.447912	17.757907	11.135374
C	11.144018	16.597926	12.833601
C	10.854473	15.650974	13.809147
H	11.628328	15.348299	14.509490
C	8.829256	14.987711	16.347611
C	7.510497	15.418529	16.550563
H	6.720412	15.038815	15.908982
C	7.208080	16.337746	17.554939
C	8.166685	16.874920	18.403904
H	7.912917	17.581611	19.186610
C	9.473740	16.459129	18.175138

C	9.815860	15.544802	17.181369
H	10.855321	15.259044	17.045407
C	10.497796	13.055879	15.462890
C	11.458115	12.652991	14.518519
H	11.434461	13.063220	13.512351
C	12.450001	11.736650	14.867394
C	12.526187	11.153103	16.128206
H	13.299250	10.439231	16.383492
C	11.558412	11.548810	17.045950
C	10.573186	12.482324	16.743755
H	9.848358	12.765294	17.502269
B	9.230989	14.022215	15.092091
S	22.810124	0.891913	13.555502
S	21.262838	1.288040	18.848179
S	20.683614	7.554985	19.333709
S	15.496364	7.826860	17.399063
S	14.562001	1.830170	16.530823
S	16.729583	1.320281	11.477887
S	22.827057	7.392443	12.451693
S	17.526332	7.647755	10.952683
F	21.488129	0.889500	12.576145
F	22.368827	-0.593372	14.114625
F	24.161466	0.814872	14.468549
F	23.336768	2.303890	12.925773
F	23.640287	0.145569	12.370416
F	21.230841	-0.289888	18.385361
F	19.631421	1.257548	19.009039
F	21.279217	2.824139	19.426508
F	22.889462	1.263092	18.798538
F	21.385466	0.806876	20.402680
F	21.131749	6.001571	19.572769
F	19.651092	7.481754	20.616243
F	20.277740	9.129817	19.148520
F	21.815306	7.709040	18.166037
F	21.823686	8.006023	20.401354
F	14.785979	6.375460	17.117520
F	15.542078	8.152419	15.790992
F	16.063008	9.339290	17.681305
F	15.371576	7.565868	19.007127
F	13.988435	8.450547	17.404894
F	13.389044	1.932675	15.377892
F	14.351990	3.411258	16.909149
F	15.634247	1.699279	17.751800
F	14.669780	0.224675	16.216911
F	13.394519	1.481832	17.609911
F	16.656586	-0.212096	12.086750
F	15.098832	1.405493	11.389349
F	16.841352	2.808313	10.764443
F	18.353491	1.185455	11.457602
F	16.720549	0.667497	9.991265
F	23.357089	5.854299	12.646859
F	22.954361	7.637121	14.061575
F	22.419169	8.968789	12.291682
F	22.821765	7.192275	10.830818
F	24.376541	7.855934	12.303864
F	16.837578	6.162711	10.830260
F	18.393019	7.343588	9.578766
F	18.134033	9.183128	11.034445
F	16.580017	8.004081	12.232902
F	16.358140	8.228448	9.992090
C	20.236195	3.443580	15.502766
C	21.043874	2.823157	14.531001
H	21.007286	3.166784	13.500048
C	21.862550	1.745729	14.869755
C	21.954567	1.251604	16.167886
H	22.606825	0.423751	16.421269
C	21.157430	1.879258	17.122687
C	20.306293	2.937008	16.814237
H	19.681595	3.368499	17.592117
C	18.742415	5.589475	16.290652
C	19.717999	6.059426	17.187514
H	20.732307	5.672277	17.130067
C	19.392280	7.009845	18.155628
C	18.112636	7.545105	18.282414
H	17.865641	8.269358	19.051015
C	17.171326	7.108858	17.352292
C	17.461583	6.161311	16.377710

H	16.688473	5.858574	15.676558
C	19.485732	5.497963	13.840906
C	20.804438	5.928959	13.638033
H	21.594554	5.549224	14.279563
C	21.106745	6.848528	12.633955
C	20.148086	7.385795	11.785107
H	20.401799	8.092699	11.002576
C	18.841059	6.969871	12.013783
C	18.499083	6.055126	13.007232
H	17.459643	5.769242	13.143095
C	17.816983	3.566332	14.725917
C	16.856454	3.163582	15.670140
H	16.879798	3.574089	16.676207
C	15.864722	2.246960	15.321415
C	15.788867	1.662724	14.060810
H	15.016610	0.947762	13.805539
C	16.756323	2.059169	13.142983
C	17.741379	2.992874	13.445065
H	18.466005	3.275986	12.686416
B	19.084236	4.532307	15.096412
S	13.371788	8.597157	3.492544
S	11.824555	8.201086	8.785194
S	11.245533	1.934145	9.270794
S	6.057482	1.662381	7.346502
S	5.123777	7.658932	6.467839
S	7.291276	8.168906	1.415011
S	13.388773	2.096704	2.388718
S	8.088103	1.841383	0.889697
F	12.049828	8.599646	2.513177
F	12.930415	10.082445	4.051626
F	14.723191	8.674318	4.405542
F	13.898479	7.185233	2.862753
F	14.202040	9.343558	2.307461
F	11.792580	9.779020	8.322363
F	10.193151	8.231602	8.946068
F	11.840970	6.665000	9.363530
F	13.451189	8.226047	8.735534
F	11.947208	8.682274	10.339685
F	11.693532	3.487591	9.509796
F	10.212911	2.007373	10.553304
F	10.839540	0.359278	9.085558
F	12.377091	1.780089	8.103040
F	12.385482	1.483128	10.338378
F	5.347019	3.112404	7.055130
F	6.099373	1.336198	5.734104
F	6.625544	0.149346	7.618829
F	5.937033	1.925760	8.950213
F	4.549866	1.038934	7.401957
F	3.950784	7.556473	5.314896
F	4.913703	6.077851	6.846196
F	6.195999	7.789868	7.688810
F	5.231543	9.264404	6.153901
F	3.956260	8.007305	7.546916
F	7.218108	9.701293	2.023770
F	5.660451	8.083787	1.326416
F	7.403072	6.680861	0.701450
F	8.915171	8.303695	1.394649
F	7.282280	8.821647	-0.071703
F	13.918808	3.634856	2.583867
F	13.516102	1.852026	3.998596
F	12.980905	0.520354	2.228687
F	13.383497	2.296870	0.767830
F	14.938278	1.633224	2.240870
F	7.399371	3.326444	0.767251
F	8.954770	2.145539	-0.484231
F	8.695750	0.305993	0.971505
F	7.141777	1.485038	2.169914
F	6.919897	1.260701	-0.070888
C	10.797773	6.044988	5.440112
C	11.605389	6.665522	4.468221
H	11.568786	6.321845	3.437278
C	12.424012	7.743134	4.806779
C	12.516022	8.237378	6.104863
H	13.168164	9.065352	6.358160
C	11.719038	7.609693	7.059759
C	10.868037	6.551739	6.751517
H	10.243529	6.120229	7.529541

C	9.303083	3.898948	6.228333
C	10.279401	3.429088	7.124442
H	11.293692	3.816150	7.066122
C	9.954317	2.479116	8.093130
C	8.674826	1.944203	8.221357
H	8.428604	1.220388	8.990604
C	7.732494	2.380379	7.292386
C	8.022043	3.327322	6.316832
H	7.248188	3.629998	5.616490
C	10.047258	3.990590	3.778374
C	11.366017	3.559773	3.575422
H	12.156103	3.939489	4.217001
C	11.668433	2.640555	2.571046
C	10.709828	2.103373	1.722085
H	10.963598	1.396690	0.939373
C	9.402777	2.519177	1.950847
C	9.060650	3.433493	2.944624
H	8.021190	3.719256	3.080581
C	8.378716	5.922422	4.663097
C	7.418395	6.325307	5.607466
H	7.442047	5.915078	6.613634
C	6.426510	7.241651	5.258592
C	6.350324	7.825196	3.997779
H	5.577263	8.539072	3.742494
C	7.318100	7.429491	3.080037
C	8.303325	6.495976	3.382232
H	9.028155	6.213008	2.623719
B	9.645526	4.956086	5.033893
S	15.531787	18.086388	6.570482
S	17.079073	17.690263	1.277808
S	17.658297	11.423312	0.792280
S	22.845538	11.151426	2.726924
S	23.779912	17.148132	3.595165
S	21.612330	17.658018	8.648101
S	15.514858	11.585857	7.674296
S	20.815555	11.330549	9.173298
F	16.853784	18.088801	7.549840
F	15.973107	19.571666	6.011361
F	14.180444	18.163437	5.657441
F	15.005148	16.674410	7.200216
F	14.701621	18.832729	7.755569
F	17.111072	19.268189	1.740623
F	18.710492	17.720753	1.116943
F	17.062694	16.154160	0.699480
F	15.452452	17.715207	1.327449
F	16.956445	18.171424	-0.276693
F	17.210164	12.976727	0.553219
F	18.690822	11.496546	-0.490253
F	18.064170	9.848479	0.977465
F	16.526606	11.269259	1.959951
F	16.518229	10.972275	-0.275369
F	23.555915	12.602835	3.008471
F	22.799843	10.825958	4.335008
F	22.278986	9.638966	2.444683
F	22.970335	11.412505	1.118871
F	24.353478	10.527747	2.721119
F	24.952868	17.045626	4.748095
F	23.989923	15.567042	3.216840
F	22.707667	17.279020	2.374185
F	23.672131	18.753625	3.909076
F	24.947394	17.496469	2.516076
F	21.685326	19.190395	8.039238
F	23.243081	17.572807	8.736637
F	21.500561	16.169985	9.361542
F	19.988422	17.792847	8.668382
F	21.621362	18.310803	10.134723
F	14.984819	13.123996	7.479133
F	15.387552	11.341186	6.064413
F	15.922752	10.009508	7.834298
F	15.520153	11.786020	9.295173
F	13.965373	11.122367	7.822125
F	21.504281	12.815603	9.295756
F	19.948884	11.634690	10.547234
F	20.207908	9.795156	9.091473
F	21.761866	10.974193	7.893086
F	21.983762	10.749851	10.133883
C	18.105719	15.534729	4.623217

C	17.298029	16.155144	5.594977
H	17.334613	15.811519	6.625930
C	16.479363	17.232580	5.256227
C	16.387343	17.726706	3.958096
H	15.735094	18.554566	3.704711
C	17.184483	17.099051	3.003297
C	18.035614	16.041295	3.311744
H	18.660314	15.609809	2.533862
C	19.599499	13.388836	3.835333
C	18.623907	12.918868	2.938488
H	17.609603	13.306027	2.995929
C	18.949631	11.968467	1.970357
C	20.229265	11.433179	1.843596
H	20.476261	10.708929	1.074993
C	21.170589	11.869454	2.773689
C	20.880321	12.816974	3.748296
H	21.653432	13.119716	4.449447
C	18.856175	13.480342	6.285087
C	17.537470	13.049343	6.487955
H	16.747350	13.429087	5.846434
C	17.235167	12.129773	7.492033
C	18.193822	11.592491	8.340874
H	17.940105	10.885602	9.123419
C	19.500842	12.008440	8.112204
C	19.842833	12.923162	7.118740
H	20.882272	13.209058	6.982889
C	20.524927	15.411971	5.400068
C	21.485465	15.814714	4.455850
H	21.462125	15.404206	3.449782
C	22.477192	16.731342	4.804571
C	22.553049	17.315576	6.065177
H	23.325300	18.030545	6.320445
C	21.585588	16.919137	6.983002
C	20.600537	15.985426	6.680920
H	19.875906	15.702320	7.439567
B	19.257673	14.445995	5.029578

S.5 References

1 McKay, A. I.; Martínez-Martínez, A. J.; Griffiths, H. J.; Rees, N. H.; Waters, J. B.; Weller, A. S.; Krämer, T; Macgregor, S. A. *Organometallics* **2018**, *37*, 3524-3532

2 Pike, S. D.; Chadwick, F. M.; Rees, N. H.; Scott, M. P.; Weller, A. S.; Krämer, T; Macgregor, S. A. *J. Am. Chem. Soc.* **2015**, *137*, 820-833

3 Chadwick, F. M.; McKay, A. I.; Martinez-Martinez, A. J.; Rees, N. H.; Krämer, T; Macgregor, S. A.; Weller, A. S. *Chem. Sci.*, **2017**, *8*, 6014-6029

4 Palatinus, L.; Chapuis, G. *J. Appl. Crystallogr.* **2007**, *40*, 786-790.

5 Sheldrick, G. M., *Acta Crystallogr. Sect. C* **2015**, *71*, 3-8.

6 Dolomanov, O. V.; Bourhis, L. J.; Gildea, R. J.; Howard, J. A. K.; Puschmann, H., *J. Appl. Crystallogr.* **2009**, *42*, 339-341.

7 Winter, G.; Waterman, D. G.; Parkhurst, J. M.; Brewster, A. S.; Gildea, R. J.; Gerstel, M.; Fuentes-Montero, L.; Vollmar, M.; Michels-Clark, T.; Young, I. D.; Sauter N. K.; Evans, G., *Acta Crystallogr. Sect. D* **2018**, *74*, 85-97.

8 Sheldrick, G. M., *Acta Crystallogr. Sect. A* **2015**, *71*, 3-8.

9 Peng, L.-M., *Micron* **1999**, *30*, 625-648.

10 Gruene, T.; Hahn, H. W.; Luebben, A. V.; Meilleur, F.; Sheldrick, G. M., *J. Appl. Cryst.* **2014**, *47*, 462-466.

11 Thorn, A.; B. Dittrich, B.; Sheldrick, G. M., *Acta Crystallogr. Sect. A* **2012**, *68*, 448-451.

12 Alvarez, S *Dalton Trans.*, **2013**, *42*, 8617-8636

13 CrystalMaker®: CrystalMaker Software Ltd, Oxford, England (www.crystalmaker.com)

14 Hutter, J.; Iannuzzi, M.; Schiffmann, F.; VandeVondele, J. *Wires Comput. Mol. Sci.* **2014**, *4*, 15-25.

15 Mercury CSD 2.0, Macrae, C. F.; Bruno, I. J.; Chisholm, J. A.; Edgington, P. R.; McCabe, P.; Pidcock, E.; Rodriguez-Monge, L.; Taylor, R.; vandeStreek, J.; Wood, P. A. *J. Appl. Crystallogr.* **2008**, *41*, 466-470.

16 VandeVondele, J.; Krack, M.; Mohamed, F.; Parrinello, M.; Chassaing, T.; Hutter, J. *Comput. Phys. Commun.* **2005**, *167*, 103-128.

17 VandeVondele, J.; Hutter, J. *J. Chem. Phys.* **2007**, *127*, 114105.

18 (a) Krack, M. *Theor. Chem. Acc.* **2005**, *114*, 145-152; (b) Goedecker, S.; Teter, M.; Hutter, J. *Phys. Rev. B:* **1996**, *54*, 1703-1710; (c) Hartwigsen, C.; Goedecker, S.; Hutter, J. *Phys. Rev. B:* **1998**, *58*, 3641-3662.

19 Perdew, J. P.; Burke, K.; Ernzerhof, M. *Phys. Rev. Lett.* **1996**, *77*, 3865-3868.

20 Grimme, S.; Antony, J.; Ehrlich, S.; Krieg, H. *J. Chem. Phys.* **2010**, *132*, 154104.

21 Bader, R. F. W. *Atoms in Molecules: A Quantum Theory*, Clarendon Press, **1994**.

22 AIMAll (Version 17.11.14), Keith, T. A. TK Gristmill Software, Overland Park KS, USA, 2017, http://aim.tkgristmill.com/.

23 NBO 6.0, Glendening, E. D.; Badenhoop, J. K.; Reed, A. E.; Carpenter, J. E.; Bohmann, J. A.; Morales, C. M.; Landis, C. R.; Weinhold, F. Theoretical Chemistry Institute, University of Wisconsin, Madison, WI, 2013, http://nbo6.chem.wisc.edu/.

24 (a) Johnson, E. R.; Keinan, S.; Mori-Sánchez, P.; Contreras-García, J.; Cohen, A. J.; Yang, W. *J. Am. Chem. Soc.* **2010**, *132*, 6498-6506; (b) Contreras-García, J.; Johnson, E. R.; Keinan, S.; Chaudret, R.; Piquemal, J.-P.; Beratan, D. N.; Yang, W. *J. Chem.Theory Comput.* **2011**, *7*, 625-632.

25 Chemcraft - graphical software for visualization of quantum chemistry computations. , https://www.chemcraftprog.com

26 Turner, M. J.; McKinnon, J. J.; Wolff, S. K.; Grimwood, D. J.; Spackman, P. R.; Jayatilaka, D.; Spackman, M. A. University of Western Australia 2017, https://hirshfeldsurface.net.
